# Ubiquitin ligase subunit FBXO9 inhibits V-ATPase assembly and impedes lung cancer metastasis

**DOI:** 10.1186/s40164-024-00497-4

**Published:** 2024-03-14

**Authors:** Liang Liu, Xiaodong Chen, Leilei Wu, Kaizong Huang, Zhenyi Wang, Yaolin Zheng, Cheng Zheng, Zhenshan Zhang, Jiayan Chen, Jiaming Wei, Song Chen, Weilin Jin, Jinfei Chen, Dongping Wei, Yaping Xu

**Affiliations:** 1 Department of Radiation Oncology, Shanghai Pulmonary Hospital, School of Medicine, Tongji University, Shanghai, 200433 China; 2 Institute of Clinical Medicine, Zhongshan Hospital, Fudan University, Shanghai, 200032 China; 3 Department of General Surgery, The First Affiliated Hospital of Wenzhou Medical University, Wenzhou, 325015 Zhejiang Province China; 4 Department of Clinical Pharmacology Lab, Nanjing First Hospital, Nanjing Medical University, Nanjing, 210006 China; 5 Medical Research Center, The First Affiliated Hospital of Wenzhou Medical University, Wenzhou, 325015 Zhejiang Province China; 6 Department of Oncology, Nanjing First Hospital, Nanjing Medical University, Nanjing, 210006 China; 7 Department of Radiation Oncology, Shanghai Proton and Heavy Ion Center, Fudan University Cancer Center, Shanghai, 200032 China; 8 Department of Radiation Oncology, Fudan University Shanghai Cancer Center, Shanghai, 200032 China; 9 Institute of Medicinal Biotechnology, Jiangsu College of Nursing, Huai’an, 223300 Jiangsu China; 10 Translational Research Institute, Henan Provincial People’s Hospital, Academy of Medical Sciences, Zhengzhou University, Zhengzhou, 450053 Henan China; 11 Institute of Cancer Neuroscience, Medical Frontier Innovation Research Center, The First Hospital of Lanzhou University, The First Clinical Medical College of Lanzhou University, Lanzhou, 730000 China; 12 Department of Oncology, The First Affiliated Hospital of Wenzhou Medical University, Wenzhou, 730000 Zhejiang Province China

**Keywords:** FBXO9, Ubiquitination, V-ATPase assembly, Migration, Cancer stemness, Metastasis

## Abstract

**Background:**

The evolutionarily conserved protein FBXO9 acts as a substrate receptor for the SKP1-cullin-1-RBX1 ubiquitin ligase and is implicated in cancer, exhibiting either tumor-suppressive or oncogenic effects depending on the specific tumor type. However, their role in lung cancer metastasis remains unclear.

**Methods:**

Lentiviral vectors carrying miRNA-based shRNA sequences for gene-specific knockdown were generated, and Lenti-CRISPR-Cas9 vectors containing gene-specific sgRNA sequences were designed. Gene overexpression was achieved using doxycycline-inducible lentiviral constructs, while gene knockdown or knockout cells were generated using shRNA and CRISPR-Cas9, respectively. Functional assays included migration, clonogenic survival assays, tumor sphere assays, and protein interaction studies using mass spectrometry, immunoprecipitation, and immunoblot analysis.

**Results:**

This study identified FBXO9 as a crucial regulator that suppresses lung cancer cell migration, tumor sphere growth and restricts metastasis. We showed that FBXO9 facilitates the ubiquitination of the catalytic subunit A (ATP6V1A) of the Vacuolar-type H^+^-ATPase (V-ATPase), resulting in its interaction with the cytoplasmic chaperone HSPA8 and subsequent sequestration within the cytoplasm. This process hinders the assembly of functional V-ATPase, resulting in reduced vesicular acidification. In contrast, depletion of FBXO9 reduced ATP6V1A ubiquitination, resulting in increased V-ATPase assembly and vesicular acidification, thus promoting pro-metastatic Wnt signaling and metastasis of lung cancer cells. Furthermore, we demonstrated the effectiveness of inhibitors targeting V-ATPase in inhibiting lung cancer metastasis in a mouse model. Finally, we established a correlation between lower FBXO9 levels and poorer survival outcomes in patients with lung cancer.

**Conclusion:**

These findings collectively elucidate the critical role of FBXO9 in regulating V-ATPase assembly and provide a molecular basis for FBXO9’s function in inhibiting lung cancer metastasis. This highlights the potential therapeutic opportunities of FBXO9 supplementation.

**Supplementary Information:**

The online version contains supplementary material available at 10.1186/s40164-024-00497-4.

## Background

Lung cancer is an extremely lethal disease, and non-small cell lung cancer (NSCLC) represents the most prevalent and deadliest subtype, accounting for approximately 80–85% of all cases [1]. Surgical resection is the primary therapeutic approach in the management of stage I-IIIA NSCLC, while platinum-based therapy commonly serves as the first-line treatment for advanced disease [2, 3]. Despite these interventions, most patients die of distant metastases, which displays a distinct propensity for infiltration into the bone, brain, and liver [4, 5]. An unfavorable prognosis can be largely attributed to the complex process of metastasis, which involves a combination of genetic and environmental factors within the in vivo microenvironment [6–8]. Therefore, the underlying mechanisms driving metastasis in lung cancer must be better understood to formulate efficacious therapeutic strategies.

Vacuolar-type H^+^-ATPase (V-ATPase) is an integral protein complex that is indispensable for maintaining acidity within various cellular compartments. This transmembrane complex functions as an ATP-dependent proton pump, thereby facilitating intracellular acidification in lysosomes, the Golgi apparatus, secretory vesicles and endosomes [9, 10]. Additionally, it aids in extracellular acidification of certain cell types [11, 12]. V-ATPase has two main constituents, namely, the cytoplasmic V1 domain and membrane-bound Vo domain, which are both involved in these processes. In mammals, the V1 domain consists of eight subunits and is responsible for ATP hydrolysis while the Vo domain consists of six subunits that help transfer H^+^ ions [9, 10]. The functionality of V-ATPases relies heavily on the correct assembly of these domains [13, 14]. The assembly process is influenced by numerous environmental signals, such as elevated glucose levels, amino acid deficiency, and various growth factors [15–17]. V-ATPase plays a considerable role in multiple cellular functions, such as autophagy, nutrient metabolism, and signaling pathway regulation, such as the Wnt, mTOR, Notch, and GPCR pathways [18, 19]. Cancer cells frequently exhibit V-ATPase dysregulation, which affects their survival, drug resistance, and metastatic abilities [19–21]. Consequently, a detailed understanding of the molecular events regulating the assembly and disassembly of V-ATPase domains could offer valuable insights for the development of therapeutic interventions.

Protein ubiquitination is key to the regulation of most cellular processes, with E3 ubiquitin ligases playing a critical role in substrate recognition and directing the covalent attachment of ubiquitin to proteins [22]. The Cullin-RING ubiquitin ligase (CRL) family is the largest group among the various ubiquitin ligase families, wherein Cullin-1-RING ubiquitin ligase (CRL1) or SCF (SKP1-cullin-1-F-box protein) is a prototypical and extensively studied member. The CRL1/SCF complex consists of Cullin-1, a scaffold protein that interacts with either RBX1 or RBX2 (RING finger proteins) at its C-terminus, thus forming an essential catalytic unit for ligase activity [23]. Cullin-1 is also associated with SKP1 (an adaptor protein) at its N-terminus. Together, they bind to each F-box protein, the substrate recognition receptor, and determine substrate specificity [23, 24]. CRL1/SCF ubiquitin ligases critically influence various physiological and pathological processes (including tumorigenesis) by enabling timely ubiquitination-dependent degradation or non-degradative ubiquitination of intracellular proteins [25–27].

The human F-box protein family consist of 69 members classified into 3 distinct subfamilies: FBXL, FBXW, and FBXO [27]. A subset of these proteins plays a crucial role in cancer development. Proteins such as SKP2, FBXL13, FBXL17, FBXL18, FBXL20, FBXO42, FBXO5, FBXO28, FBXL10, and FBXO7 highlight their significance in promoting oncogenesis through ubiquitylation-induced degradation of tumor suppressors [28–31]. Conversely, several other F-box proteins including FBXW2, FBXW7, FBXL2, FBXL3, FBXL4, FBXL5, FBXL14, FBXL19, FBXO1, and FBXO4 are implicated in tumor suppression as they facilitate the selective degradation of oncogenic proteins [28–31]. Notably, specific F-box proteins such as β-TRCP1 and β-TRCP2 can either promote or hinder tumor growth depending on the specific tumor and cellular context [32]. FBXO9 (also known as FBX9 or NY-REN-57) belongs to the FBXO subfamily and is recognized as the substrate recognition subunit of the CRL1/SCF E3 ligase complex [33]. FBXO9 tends to exhibit oncogenic behavior in conditions such as multiple myeloma and hepatic carcinoma [34, 35], although its role in various diseases (including cancer) is largely overlooked. However, it inhibits tumorigenesis in acute myeloid leukemia [36]. These findings suggest that the impacts of FBXO9 on cancer development may vary depending on the exact type of cancer or cellular context. However, the role of FBXO9 in the metastasis of lung cancer remains unclear.

This study investigated the tumor-suppressive functions of FBXO9 in lung cancer. FBXO9 plays a crucial role in inhibiting lung cancer cell migration, tumor sphere growth, and metastasis. Our findings highlight the significant role of FBXO9 in the regulation of V-ATPase activity and provide insights into the molecular mechanisms underlying the suppression of lung cancer metastasis.

## Materials and methods

### Antibodies and reagents

All antibodies and key reagents used in this study are listed in Supplementary Table 1.

### Cell culture

889DTC cells (*Kras*^G12D^/*Trp53*^−/−^) [37] were provided by Dr. Monte M. Winslow from the Stanford University School of Medicine. The A549 and H1299 human lung cancer cell lines were obtained from the Cell Bank Type Culture Collection of the Chinese Academy of Sciences. The cancer cells were cultured in RPMI1640 medium supplemented with 10% fetal bovine serum (FBS), penicillin (100 U/ml), and streptomycin (100 µg/ml). HEK293T cells were cultured in Dulbecco’s Modified Eagle’s Medium (DMEM) supplemented with 10% FBS and 2 mM glutamine. All cells were maintained at 37 °C with 5% CO_2_ in a humidified atmosphere.

### Plasmid construction

The cDNAs of mouse Fbxl3, Fbxl5, Fbxl8, Fbxo9, Atp6v1a, Atp6v1b2, Atp6v1c1, Atp6v1c2, Atp6v1d, Atp6v1e1, Atp6v1f, Atp6v1g1, human FBXO9, and ATP6V1A were inserted into different plasmid vectors. This involved techniques such as sub-cloning or polymerase chain reaction (PCR)-based site-directed mutagenesis. The plasmid vectors included the Tet-On lentiviral vector with N-terminal FLAG and pcDNA3.1 vectors modified with N-terminal HA and FLAG tags. Further information regarding these plasmids, which were stored in the Wei lab, can be provided upon request.

The SplashRNA online tool (http://splashrna.mskcc.org/) [38] was used to design microRNA-based shRNA sequences for generating gene-specific lentiviral shRNA vectors. The specific shRNAs were encoded in 97-mer oligonucleotides, which can be found in Supplementary Table 2. Following an established protocol [38, 39], two doxycycline-inducible shRNAs targeting different regions of each gene and a control shRNA targeting the LacZ gene (shLac), were constructed using the LT3GEPIR (pRRL) vector [39].

To generate Lenti-CRISPR-Cas9 vectors containing the target sgRNA, we designed gene-specific sgRNA sequences using the online CRISPR design tool available at http://www.deephf.com. The sgRNA oligos were subsequently integrated into the LentiCRISPR v2 vector (Addgene) using standard protocols. The following sgRNA sequences were employed: non-targeting sgCtrl (GAAGGGCATCGACTTCAAGG), human sgFBXO9 (CCCCCCTCAGGCAGAAGCTG), and mouse sgFbxo9 (CATGACTGGAGCCTACACCT).

### Lentiviral transduction

Lentiviral particles were generated by co-transfecting the lentiviral transfer vector, psPAX2 packaging plasmid, and pMD2.G envelope plasmid into HEK293T cells. The transfection was performed using Lipofectamine 2000 (Thermo Fisher) according to a standard protocol. Following transfection, the lentiviral supernatants were filtered using a 0.45-µm filter membrane before being applied to the desired cells.

### Gene overexpression

To achieve stable and regulated gene overexpression, the doxycycline-responsive lentiviral expression system was utilized. Full-length Fbxl3, Fbxl5, FBXL8, Fbxo9, and FBXO9 and mutant variants of Fbxo9 and FBXO9 with F-box domain deletions were introduced into cancer cells using lentiviral constructs. Following transduction, puromycin-resistant cells were treated with doxycycline for 72 h in preparation for subsequent experiments. For transient expression, plasmid constructs were transfected into HEK293T cells using Lipofectamine 2000.

### Gene knockdown or knockout cells

To knock down human FBXO9 or mouse Fbxo9, cancer cells were transduced with lentivirus carrying shRNA specifically targeting the respective gene. This lentivirus also contained a puromycin resistance marker for selection. Subsequently, the puromycin-resistant cells were treated with doxycycline for 72 h. Furthermore, for the knockdown of human FBXO9, cancer cells were transiently transfected with siRNA oligonucleotides using Lipofectamine RNAiMAX (Thermo Fisher), following the manufacturer’s guidelines. The knockdown efficacy was assessed using quantitative-PCR and immunoblot analysis.

To generate isogenic single clones lacking the target gene, cancer cells were transduced with lentivirus encoding Cas9/sgRNA and co-expressing a puromycin resistance marker. The puromycin-resistant cells were then plated as single cells using the limiting dilution technique in 96-well plates. After 2 weeks of incubation, the resulting single clones were evaluated for the knockout phenotype through immunoblot analysis and Sanger sequencing of the genomic DNA region encompassing the cleavage site.

### Migration assay

We seeded 4 × 10^4^ cancer cells in 100 µl of serum-free medium into the upper chamber of 8 μm pore-size transwell insert membranes (Corning). The lower chamber was filled with medium containing 10% FBS. After incubation for 6–24 h, the non-migrating cells on the upper surface of the insert were removed with a cotton swab. The insert was then fixed with methanol for 10 min, rinsed with PBS, and stained with 0.5% crystal violet blue for 15 min. After thorough washing, the migrated cells were examined, photographed, and counted under a microscope.

### Clonogenic survival assay

Cell colonies were prepared by seeding cells into three 60-mm dishes at a density range of 500–1000 cells per well. The dishes were then incubated for 7–10 days. After incubation, the cell colonies were fixed and stained using a solution containing 50% methanol, 10% acetic acid, and 0.25% Coomassie brilliant blue G250. Images of the resultant colonies were captured via light microscopy for visual analysis.

### Tumor sphere assays

Cancer cells were digested with 0.05% Trypsin to generate a cell suspension. Subsequently, approximately 2 × 10^3^ cells were cultured per well in 24-well plates (Corning) with ultra-low attachment, following established protocols [40].

### Mass spectrometry analysis

To study protein–protein interactions, cells expressing the FLAG-FBxo9-ΔF protein were grown in 10 cm dishes until they reached approximately 80% confluency. The cells were then washed twice with PBS (pH 7.4). To enhance the preservation of protein-protein interactions, the cells were treated with 2 mM of the cross-linker dithiocarbamate (DSP) for 30 min at room temperature. The cross-linking reaction was neutralized by incubating the cells with 20 mM Tris-HCl (pH 7.5) for 15 min at room temperature. After a single wash with PBS, the cells were lysed using NP-40 buffer containing a cocktail of protease inhibitors (Roche). The lysates were collected by scraping and pipetting, followed by centrifugation at 10,000 ×*g* for 10 min. The resulting supernatants were then subjected to affinity purification using anti-FLAG M2 beads (Sigma). Following five washes with PBS, the purified protein complexes were analyzed by mass spectrometry (MS).

### Immunoprecipitation and immunoblot analysis

Whole-cell extracts were obtained using NP40 lysis buffer supplemented with a protease inhibitor. Equivalent protein concentrations were used for immunoprecipitation. This process was facilitated using either anti-FLAG M2 beads (Sigma) or Pierce Anti-HA Magnetic Beads (Thermo Fisher). The incubation was set to one hour at room temperature. Following immunoprecipitation, the resulting immune complexes underwent five rounds of washing with TBST buffer (20 mM Tris pH 7.5, 150 mM NaCl, and 0.05% Tween 20). Elution of the immune complexes was achieved by boiling in 1x SDS sample buffer, followed by separation using SDS-PAGE. Subsequently, the separated proteins were transferred onto a blotting membrane and probed with the relevant antibodies.

### Ubiquitination assay

HEK293T cells were co-transfected with 6×His-Ubiquitin and other relevant constructs. After 48 h, the cells were lysed using buffer A, which contained 6 M guanidinium-HCl (pH 8.0), 0.1 M Na2HPO4/NaH2PO4, and 10 mM imidazole. The lysates were then sonicated and incubated with nickel-nitrilotriacetic acid (Ni-NTA) beads (Qiagen) at room temperature for 3 h. The pulled down proteins were washed twice with buffer A, twice with buffer A/TI (buffer A:buffer TI = 1:3), and once more with buffer TI. The ubiquitinated proteins were eluted by boiling in 2x SDS sample buffer and then separated by SDS-PAGE. Finally, the proteins were analyzed by immunoblot using the specified antibodies.

### Protein half-life assay

In the experimental protocol, cycloheximide (CHX) was added to the cell culture medium at a final concentration of 50 µg/mL and maintained at 37 °C. The cells were then harvested at specific time points, and the target proteins were extracted and identified through immunoblotting. The target proteins were quantified using ImageJ software (version 1.52), and the results were displayed using GraphPad Prism software (version 9). In all CHX assays, the quantified proteins were normalized relative to the loading control.

### LysoTracker staining

Live cells were stained with the LysoTracker Red DND-99 probe (Thermo Fisher) to visualize lysosomes, following a protocol as described before [41]. Subsequently, images of the stained cells were captured using an inverted fluorescence microscope. To quantify the fluorescence intensity of the LysoTracker stain, Fiji/ImageJ software was utilized.

### Subcellular fractionation

After reaching 80–90% confluence, the cells were harvested for subcellular fractionation using the Minute Plasma Membrane Protein Isolation and Cell Fractionation Kit (Invent Biotechnologies), following the manufacturer’s instructions. The resulting subcellular fractions were combined with SDS loading buffer and boiled at 98 °C for 10 min before conducting the immunoblotting analysis.

### Animal experiments

Female athymic nude mice (aged between 5 and 7 weeks) were obtained from the Shanghai Laboratory Animal Center (SLAC) and had ad libitum access to both food and water. The Committee on the Use and Care of Animals at Shanghai Pulmonary Hospital authorized all procedures involving mice. To examine the in vivo metastasis effects of Fbxo9 overexpression in 889DTC cells and FBXO9 suppression in H1299 cells, we introduced 1 × 10^5^ 889DTC cells (expressing EGFP and Fbxo9) and 1 × 10^6^ H1299 cells (stably expressing shLacZ and shFBXO9) into the lateral tail veins of the nude mice (*n* = 6 per group). To evaluate the impact of FBXO9-mediated inhibition of ATP6V1A ubiquitination on lung metastasis in vivo, 1 × 10^6^ H1299 cells expressing V1A-23 aa (a peptide designed to inhibits ATP6V1A ubiquitination) were administered to mice via the tail vein (*n* = 6 mice per group). To assess the effectiveness of V-ATPase inhibition in preventing metastasis, two V-ATPase inhibitors, bafilomycin A1 (BAF) and lansoprazole (LAN), were employed. Mice injected with 1 × 10^5^ 889DTC cells received intraperitoneal treatment involving either a vehicle (5% DMSO in corn oil), BAF (1 mg/kg), or LAN at doses of 20 mg/kg or 40 mg/kg (*n* = 5–6 mice per group). These treatments were administered every other day for a total of five consecutive treatments over a 21-day period. To determine the extent of cancer cell metastasis in the lung tissue, fluorescence microscopy, bioluminescent imaging, and H&E staining were conducted at the study endpoint (at 2–3 weeks for 889DTC, and 6 weeks for H1299 and A549, respectively).

### Database analysis

The RNA-seq data and clinical information on lung adenocarcinoma (LUAD) and lung squamous cell carcinoma (LUSC) were acquired from The Cancer Genome Atlas (TCGA) public database, which is accessible at https://portal.gdc.cancer.gov. Additionally, the GSE31210 dataset was obtained from the Gene Expression Omnibus (GEO) public database, which is accessible at https://www.ncbi.nlm.nih.gov/geo/. These datasets were utilized to investigate the expression levels of FBXO9 and the prognostic significance of FBXO9 in lung cancer.

### Statistical analysis

All in vitro experiments were performed at least twice unless explicitly indicated otherwise. Statistical examinations were conducted using GraphPad Prism 9.0 (La Jolla, CA, USA) and included various suitable tests, such as Student’s t-test, ANOVA, and Kaplan–Meier analysis, each chosen in accordance with the specific needs of each test. Data were delineated as the mean ± standard deviation (SD), with statistical significance determined by the thresholds of *P < 0.05, **P < 0.01, and ***P < 0.001.

## Results

### FBXO9 exerts anti-metastatic activity in lung cancer cells

**Fig. 1:**
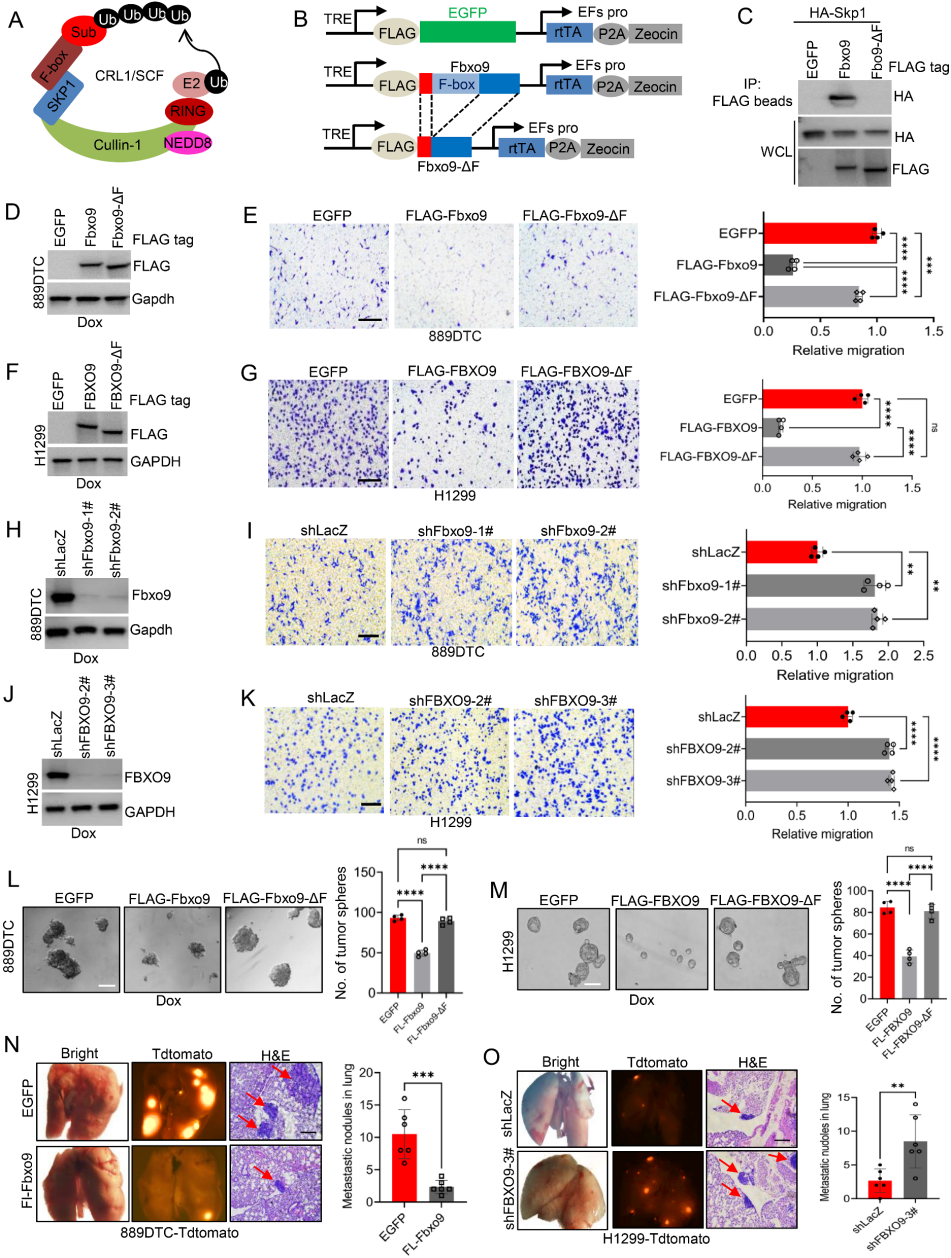
Suppressive role of FBXO9 in migration, tumor sphere formation and metastasis of lung cancer cells. **A** Schematic diagram illustrating the role of F-box proteins in CRL1/SCF ubiquitin ligase. **B** Fbxo9 expression, including that of the Fbxo9-ΔF mutant lacking the F-box domain, is controlled using the Tet-On system. **C** Co-immunoprecipitation assay identifying the binding capacity between Fbxo9-ΔF and the Skp protein. **D** Immunoblot analysis of 889DTC cells expressing Fbxo9 and Fbxo9-ΔF, with Gapdh as a loading control. **E** Representative images and quantitative results of migrated 889DTC cells expressing either Fbxo9 or Fbxo9-ΔF(*P* < 0.001). **F** Immunoblot analysis of H1299 cells expressing FBXO9 and FBXO9-ΔF, with GAPDH serving as a loading control. **G** Representative images (left) and quantitative results (right) of migrated H1299 cells expressing FBXO9 or FBXO9-ΔF (*P* < 0.0001). **H** Immunoblot analysis of Fbxo9 knockdown in 889DTC cells using shRNA, with Gapdh as a loading control. **I** Representative images (left) and quantitative results (right) of migrated 889DTC cells with Fbxo9 knockdown (*P* < 0.01). **J** Immunoblot analysis confirming FBXO9 knockdown in H1299 cells using shRNA. **K** Representative images (left) and quantitative results (right) of cell migration in H1299 cells with FBXO9 depletion (*P* < 0.0001). **L** Impact of Fbxo9 knockdown on tumor sphere formation in 889DTC cells (*P* < 0.0001). **M** Impact of FBXO9 knockdown on tumor sphere formation in H1299 cells (*P* < 0.0001). **N** Ectopic expression of Fbxo9 reduces lung cancer cell metastasis in vivo. Nude mice were injected with 889DTC cells expressing Fbxo9 via the tail vein (*n* = 6 each group). After two weeks, lung tissues were analyzed. Fluorescence microscopy detected the presence of 889DTC-Tdtomato cells, as indicated by orange fluorescence (left). The number of metastasis nodules was quantified based on fluorescence and statistically analyzed (right) (*P* < 0.001). **O** Downregulation of FBXO9 promotes lung cancer cell metastasis in vivo. H1299-Tdtomato cells with stable FBXO9 knockdown were injected into the tail vein of nude mice (*n* = 6 each group). After 6 weeks, lung tissues were collected for fluorescence microscopy (left) and H&E staining (left). Metastasis nodules were quantified via fluorescence microscopy and statistically analyzed (right) (*P* < 0.01). All error bars represent the mean ± SD. *P < 0.05, **P < 0.01, ***P < 0.001

We performed a survival analysis using the KM plotter online tool [42] to explore the potential association between F-box proteins (Fig. 1A) and overall survival (OS) in lung cancer patients. Our preliminary data indicated that the enhanced expression of certain F-box genes (including FBXL3, FBXL5, FBXL8, and FBXO9) exerted a favorable influence on OS (Supplementary Fig. S1A). Building on these observations, we integrated the tetracycline (Tet)-inducible gene expression system to regulate the specific F-box gene cDNA (as depicted in Supplementary Fig. S1B) in 889DTC cells (*Kras*^G12D^ /*Trp53*^−/−^), which are known for their high metastatic potential [37]. We performed a cell migration assay after confirming the successful expression of these F-box genes by immunoblot analysis (Supplementary Fig. S1C). Overexpression of Fbxo9 significantly reduced cancer cell migration compared with the overexpression of Fbxl3, Fbxl5, or Fbxl8 (Supplementary Fig. S1D and E). However, its overexpression had only a minimal effect on clonogenic survival (Supplementary Fig. S1F and G). These findings prompted further investigation into the biological role of Fbxo9 in various cancer cell lines.

We generated a mutated variant of Fbxo9 (known as Fbxo9-ΔF) that lacks the F-box domain (Fig. 1B). The Tet-On gene expression system was used to control the expression of intact Fbxo9 and Fbxo9-ΔF within the cells (Fig. 1B). Our IP assay confirmed that unlike full-length Fbxo9, Fbxo9-ΔF cannot interact with Skp protein (an adaptor subunit of CRL1/SCF ubiquitin ligase) (Fig. 1C). This indicates that Fbxo9-ΔF could not facilitate the assembly of the E3 ligase complex. Subsequently, we introduced full-length Fbxo9 and Fbxo9-ΔF into 889DTC cells and induced their expression using doxycycline (Fig. 1D). Cells expressing full-length Fbxo9 exhibited a significant reduction in migration compared with those expressing Fbxo9-ΔF (Fig. 1E). Similar results were obtained when overexpressing full-length FBXO9 and FBXO9-ΔF in A549 and H1299 lung cancer cells (Fig. 1F and G, Supplementary Fig. S2A-E). Conversely, the depletion of Fbxo9 or FBXO9 using shRNA in 889DTC or H1299 cells significantly increased cancer cell migration (Fig. 1H-K) but had minimal effects on the survival of lung cancer cells (Supplementary Fig. S3A and B). Overall, our observations strongly suggested that FBXO9 serves as a suppressor of lung cancer cell migration but had a limited impact on cancer cell survival.

F-box proteins are implicated in the modulation of cancer stem cell characteristics and play a crucial role in cancer metastasis [28]. The introduction of full-length Fbxo9 significantly reduced the number and size of spheres in 889DTC cells, whereas Fbxo9-ΔF had minimal effect (Fig. 1L). Similar outcomes were observed in the H1299 cell line when FBXO9 and FBXO9-ΔF were overexpressed (Fig. 1M). These findings strongly indicate a significant correlation between FBXO9 and the stem cell-like characteristics of lung cancer cells.

We conducted experiments on mice to study how altering Fbxo9 expression in 889DTC cells affects lung metastasis. We injected the cells into the mice’s tail veins and found that mice with increased Fbxo9 expression had a significantly lower number of metastatic nodules in their lungs compared with that of the control group (Fig. 1N). This indicates that higher Fbxo9 expression can suppress lung metastasis. In another experiment, we introduced H1299 cancer cells with FBXO9 knockdown through tail vein injection. After six weeks, we noticed a noticeable increase in the number of lung metastatic nodules in the FBXO9 knockdown group (Fig. 1O). This result further reinforces the significance of FBXO9 reduction in promoting lung cancer cell metastasis relative to the control group. Collectively, our findings provide evidence that FBXO9 functions as an inhibitor and effectively suppresses lung cancer cell migration, tumor sphere growth, and overall metastasis in cell cultures and mouse models.

#### FBXO9 interacts with V-ATPase

**Fig. 2:**
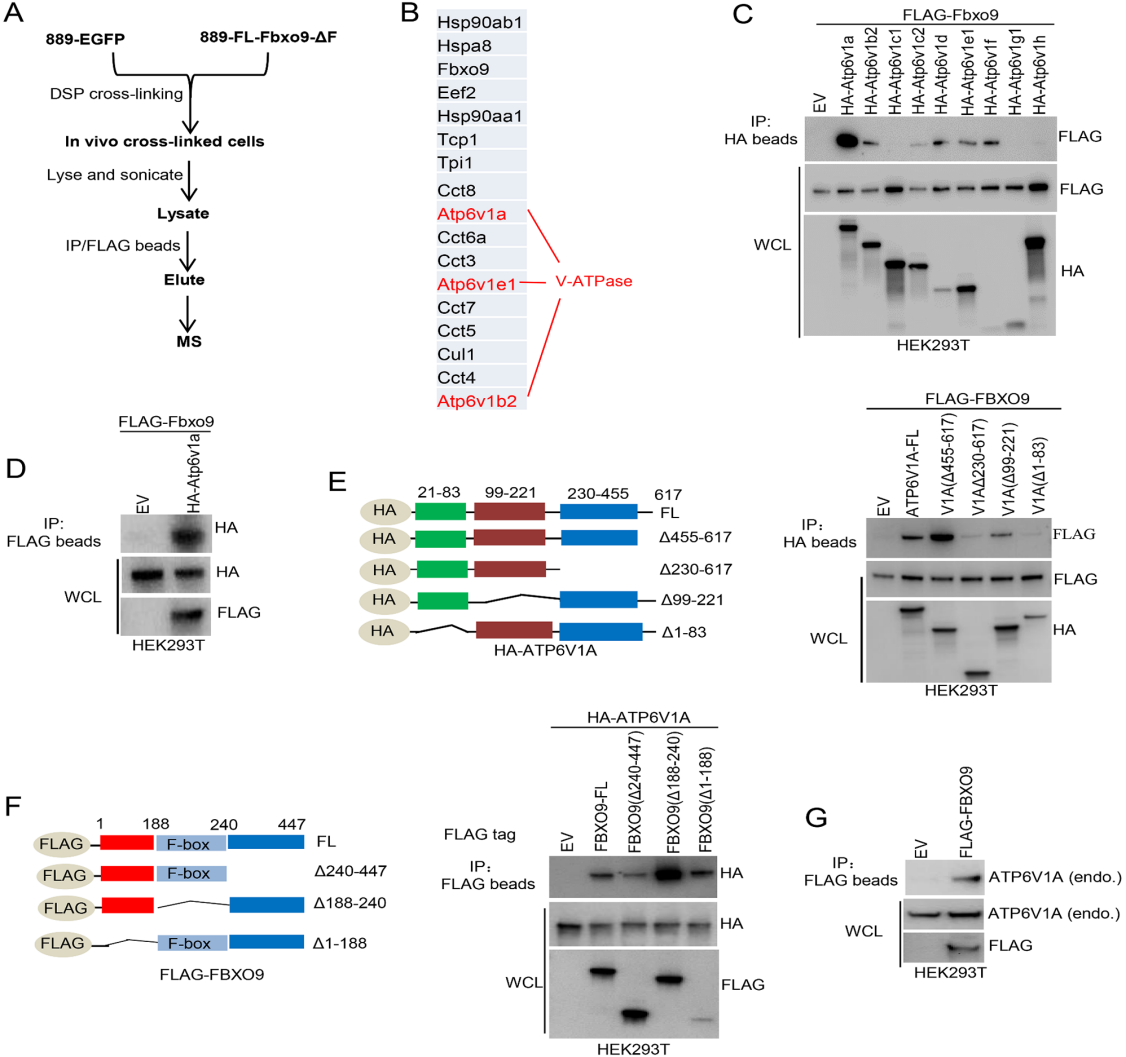
FBXO9 is closely associated with V-ATPase. **A** Workflow diagram representing the FBXO9 interactor search process using MS. **B** List of top proteins identified as potential binders to FBXO9. **C** Co-IP of different subunits (HA-tagged) of the V-ATPase V1 domain. The V-ATPase V1 domain subunits were immunoprecipitated using anti-HA beads, and the presence of bound FLAG-Fbxo9 was detected using anti-FLAG antibodies. **D** Co-IP of FLAG-Fbxo9 with HA-Atp6v1a in HEK293T cells. FLAG-Fbxo9 was immunoprecipitated using anti-FLAG antibodies, and the presence of bound HA-Atp6v1a was detected using anti-HA antibodies. **E** Schematic diagram illustrating the design of full-length HA-tagged ATP6V1A (FL) and a series of truncated, overlapping constructs (left). HEK293T cells were co-transfected with these constructs, followed by IP assay using anti-HA beads. Immunoblots were then probed with both anti-FLAG and anti-HA antibodies to detect the expression of the constructs (right). **F** Schematic diagram demonstrating the design of full-length FLAG-tagged FBXO9 (FL) and multiple truncated, overlapping constructs. HEK293T cells were co-transfected with these constructs and subsequently immunoprecipitated with anti-FLAG beads. Immunoblotting was conducted using anti-HA and anti-FLAG antibodies to detect the expression of the constructs (right). **G** Co-IP of the endogenous ATP6V1A with ectopically expressed FLAG-FBXO9 was conducted in HEK293T cells

We examined the interacting proteins of FBXO9 to elucidate the mechanism underlying the anticancer effects of FBXO9. We established a stable 889DTC cell line expressing a FLAG-tagged Fbxo9-ΔF. Subsequently, we treated the cells with DSP, a chemical crosslinking agent, and performed IP assay and MS to identify the proteins associated with Fbxo9 (Fig. 2A). Remarkably, the absence of the Skp1 binding domain in Fbxo9-ΔF hindered its association with Cullin-1 and the catalytic RBX RING finger subunit, potentially causing substrate entrapment. Consequently, we successfully identified numerous proteins that potentially associate with Fbxo9. The top protein candidates were three members of the heat shock protein family (Hsp90AA1, Hsp90AB1, and HspA8), three subunits of the V-ATPase peripheral V1 domain (Atp6v1a, Atp6v1b2, and Atp6v1f), and all subunits of the TRiC cytosolic chaperonin tailless complex polypeptide one ring complex (Cct1-Cct8) (Fig. 2B). The link between V-ATPase and cancer metastasis progression and drug resistance has been previously established [20, 43]. Therefore, we focused on the constituents of the V-ATPase V1 domain. We hypothesized that that FBXO9 might regulate V-ATPase activity and cancer metastasis through V1 domain ubiquitination. This was tested by transiently transfecting Fbxo9 and separating the V1 domain subunits into HEK293T cells, followed by performing an IP assay to examine the interaction between Fbxo9 and the V1 domain. Immunoblotting revealed that Fbxo9 co-immunoprecipitated with Atp6v1a, while other subunits of the V1 domain exhibited minimal affinity for Fbxo9 (Fig. 2C). Atp6v1a was readily pulled down when Fbxo9 was used as bait (Fig. 2D). This indicated the strong interaction between Fbxo9 and Atp6v1a.

We subsequently examined the interaction between the human proteins ATP6V1A and FBXO9. We created a series of deletion mutants for ATP6V1A (Fig. 2E) to identify the exact region of ATP6V1A involved in binding to FBXO9. Each of these constructs was individually cotransfected with the FBXO9 construct into HEK293T cells. Subsequent co-IP experiments indicated that amino acid regions 1–83 (1–83 aa) and 230–617 (230–617 aa) play crucial roles in the interaction with FBXO9 (Fig. 2E). A similar approach was employed to map other domains by generating deletion mutants of FBXO9 (Fig. 2F, left). The IP assay emphasized that the C-terminal domain (240–447 aa) of FBXO9 significantly contributed to the interaction with ATP6V1A (Fig. 2F, right). We transfected HEK293T cells with FLAG-FBXO9 and performed IP to detect endogenous ATP6V1A to strengthen our findings. Ectopically expressed FLAG-FBXO9 pulled down a substantial amount of endogenous ATP6V1A compared with the negative control (Fig. 2G). Collectively, these results confirm the interaction between FBXO9 and ATP6V1A, suggesting that FBXO9 may play a role in the function and regulation of V-ATPase.

### FBXO9 promotes non-degradative ATP6V1A ubiquitination

**Fig. 3:**
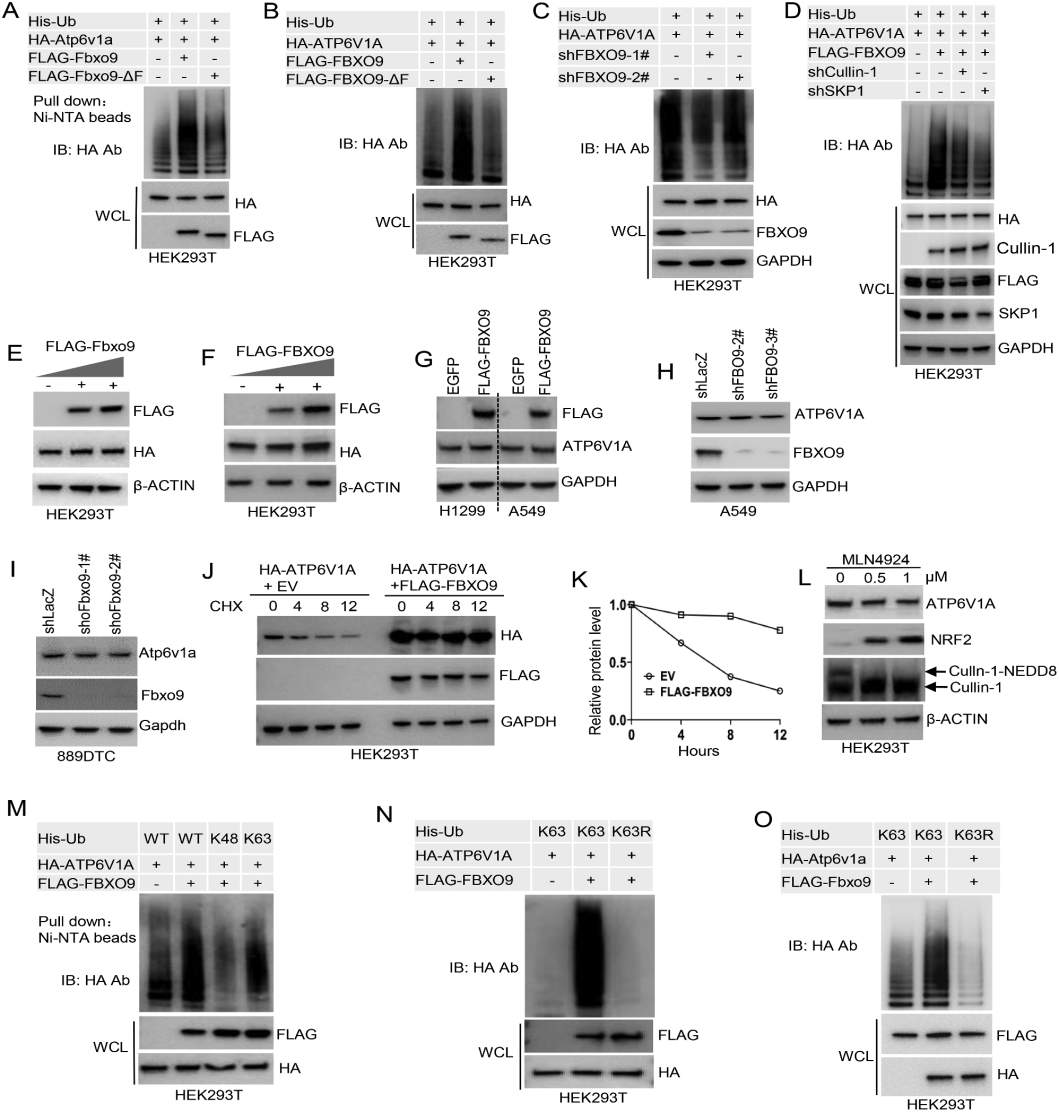
FBXO9 promotes non-degradative ubiquitination of ATP6V1A. **A**-**C** Ubiquitination assays in HEK293T cells were performed under different conditions. Co-transfection with HA-Atp6v1a (A) or HA-ATP6V1A (B) and His-Ub, along with Flag-FBXO9 after knockdown of FBXO9 using independent shRNAs (shFBXO9-2# and-3#) (C). Samples recovered with Ni-NTA (top) and whole cell lysate samples (bottom) were subjected to blotting against anti-HA or Flag as indicated. **D** Involvement of SKP1 and Cullin-1 in FBXO9-mediated ATP6V1A ubiquitination. HEK293T cells were transfected with His-Ub plasmids and shRNA targeting SKP1 or Cullin-1. Cell lysates were subjected to Ni-NTA bead pulldown, and the precipitates were analyzed by immunoblotting. **E-G** Effect of FBXO9 overexpression on exogenous (E, F) and endogenous (G) ATP6V1A protein levels. Cells were co-transfected with specific plasmids, and immunoblotting was carried out 48 h after transfection. **H, I** Effect of FBXO9 knockdown on ATP6V1A protein levels. A549 cells (H) and 889DTC cells (I) were depleted of FBXO9 or Fbxo9 using shRNA, and immunoblotting was performed. **J, K** Impact of FBXO9 expression on ATP6V1A half-life. HEK293T cells were transfected with FLAG-FBXO9 and subsequently treated with CHX for specific time periods, followed by immunoblotting analysis (J). The density of the ATP6V1A band was quantified to determine the ATP6V1A half-life (K). **L** Effect of neddylation inhibition on the ATP6V1A protein level in HEK293T cells. Cells were treated with MLN4924 for 12 h to inactivate the CRL ubiquitin ligase. Cell lysates were then analyzed by immunoblotting. **M–O** Ubiquitination of HA-ATP6V1A1 was measured in cells co-expressing FLAG-FBXO9 and different forms of His-Ub, as described in (A-C).

We investigated whether FBXO9 could promote ATP6V1A ubiquitination using a histidine-based ubiquitination assay since FBXO9 is a potential substrate recognition element in CRL1/SCF ubiquitin ligase [34, 35]. As anticipated, the ectopic expression of FBXO9 markedly augmented ATP6V1A ubiquitination. In contrast, the FBXO9-ΔF variant that lacks the essential F-box domain required for complex formation with CRL1/SCF had minimal impact on ATP6V1A ubiquitination (Fig. 3A and B). We reduced the expression of endogenous FBXO9 using shRNA to confirm the involvement of FBXO9 in ATP6V1A ubiquitination. This reduction substantially decreased ATP6V1A ubiquitination (Fig. 3C). We then selectively inhibited the expression of Cullin-1 and SKP1 (scaffold and adaptor proteins of the CRL1/SCF complex, respectively) to gain further insight into the mechanism of FBXO9. The decreased expression of these proteins also resulted in reduced FBXO9-mediated ATP6V1A ubiquitination (Fig. 3D). Taken together, these findings strongly suggest that ATP6V1A is a newly identified substrate of the CRL1/SCF^FBXO9^ E3 ligase.

We manipulated the expression of FBXO9 in HEK293T and cancer cells via gene overexpression (Fig. 3E-G) and RNA interference (Fig. 3H and I) to determine the implications of FBXO9-mediated ATP6V1A ubiquitination. The ATP6V1A levels remained largely unaffected regardless of the changes in FBXO9 expression (Fig. 3E-I). Furthermore, ectopic expression of FBXO9 did not increase ATP6V1A degradation in the CHX chase assay, which measures protein turnover (Fig. 3J and K). Additionally, treating cells with MLN4924 (a neddylation inhibitor that deactivates CRL ubiquitin ligases) did not cause significant accumulation of ATP6V1A within cells, although it increased the protein expression of NRF2 (a known substrate of CRL) (Fig. 3L). These findings suggest that the role of FBXO9 in ATP6V1A ubiquitination may not directly affect its expression or protein turnover.

Polyubiquitination typically results from the formation of ubiquitin chains at lysine (K) 48 or K63. K48-linked polyubiquitination mainly signals proteasomal degradation, while K63-linked polyubiquitination often determines protein location, activity, or complex formation [44, 45]. We performed ubiquitination assays using Ub-K48 and Ub-K63 ubiquitin mutants to investigate ATP6V1A polyubiquitination in relation to K48- or K63-linked ubiquitin chains. FBXO9 enhanced K63-linked polyubiquitination of ATP6V1A in HEK293T cells, but not K48-linked polyubiquitination with K48 His-Ub (Fig. 3M). Similarly, co-transfection of HEK293T cells with the Ub-K63R ubiquitin mutant significantly reduced ATP6V1A ubiquitination (Fig. 3N). These findings were also observed in Atp6v1a cells overexpressing Fbxo9 (Fig. 3O). These findings provide evidence that FBXO9 plays a significant role in the K63-linked polyubiquitination of ATP6V1A, suggesting its non-degradative function in cellular processes.

### ATP6V1A ubiquitination by FBXO9 suppresses lung cancer cell migration and tumor sphere growth and restricts in vivo metastasis

**Fig. 4:**
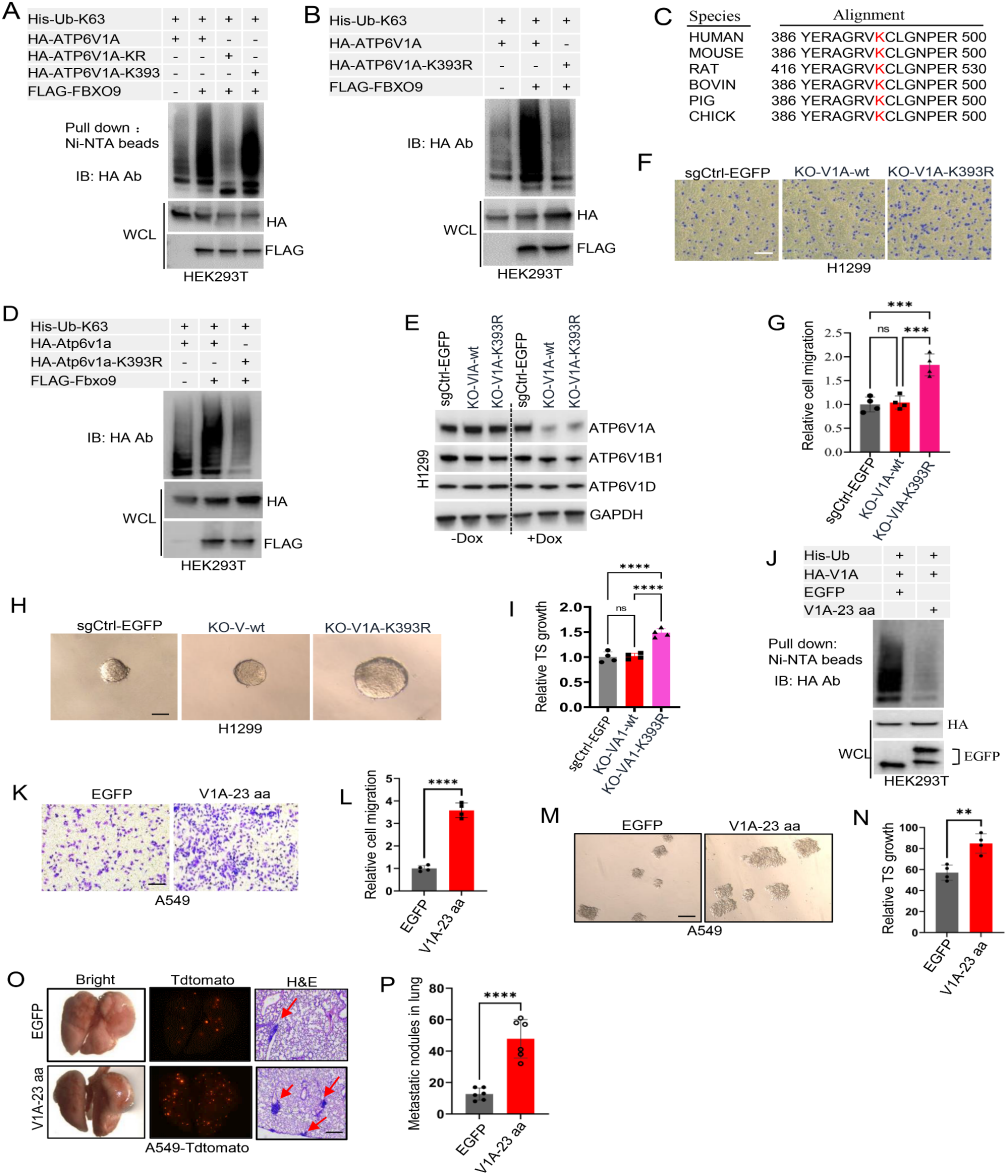
FBXO9-mediated ATP6V1A ubiquitination impairs metastasis of lung cancer cells. **A**, **B** Ubiquitination assays were performed in HEK293T cells by co-transfection with full-length HA-ATP6V1A, HA-ATP6V1A-KR (lysine residues removed), HA-ATP6V1A-K393 (K393 reintroduced into the ATP6V1A-KR mutant), or HA-ATP6V1A-K393R (single lysine substitution mutant), in the presence or absence of FLAG-FBXO9. After 48 h, cells were lysed, followed by Ni-NTA bead pull-down and immunoblotting for HA-ATP6V1A. **C** Alignment of ATP6V1A K393 conservation across species. **D** Ubiquitination analysis of K393 in HA-Atp6v1a by co-expressing Flag-Fbxo9 and His-Ub with indicated HA-Atp6v1a constructs (WT, K393, or K393R) in HEK293T cells. Cells were lysed after 48 h, followed by Ni-NTA bead pull-down and immunoblotting for HA-Atp6v1a. **E** V-ATPase complex reconstitution was achieved in H1299 cells by introducing sgRNA-resistant ATP6V1A (wild-type or K393R mutant) using a Tet-Off system. CRISPR was then used to deplete endogenous ATP6V1A, allowing the V-ATPase complex to be reconstituted. Immunoblot analysis confirmed comparable expression levels of the added ATP6V1A and endogenous subunits ATP6V1B and ATP6V1D1, in the experimental group compared to the control cells, after 48 h of doxycycline treatment. **F–I** Recombinant cell lines H1299-KO-V1A-wt, H1299-KO-V1A-K393R, and H1299-EGFP-sgCtrl were used in a transwell migration assay (F, G) and tumor sphere formation assay (H, I) to evaluate the effects of ATP6V1A ubiquitination on cell migration and tumor sphere growth(*P* < 0.001). **J–N** To assess the effects of ATP6V1A ubiquitination on cell migration and tumor sphere growth, V1A-23aa was introduced into A549 cells to inhibit ATP6V1A ubiquitination (J) which was followed by transwell migration assay (K, L) and tumor sphere formation assay (M, N) to evaluate the impact on cell migration and tumor sphere growth, respectively (*P* < 0.001). **O, P** Impact of ATP6V1A ubiquitination inhibition by FBXO9 on in vivo lung metastasis was assessed. A549 cells expressing V1A-23 aa were injected into mice via the tail vein. After 6 weeks, the lungs of the mice were harvested for examination and H&E staining. Metastasis nodules were quantified and statistically analyzed using fluorescence microscopy. Error bars represent the mean ± SD. ****P < 0.0001 indicates the significance level

We used a mutagenesis approach to determine the specific site(s) of FBXO9-mediated ATP6V1A ubiquitination. We mutated ATP6V1A so that all lysine residues were replaced with arginine (ATP6V1A-KR). We also generated a series of overlapping ATP6V1A mutants (Supplementary Fig. S4A). The mutants overlapping with ATP6V1A-KR (ATP6V1A-KR2, ATP6V1A-KR3, and ATP6V1A-KR4) showed partial ubiquitination upon FBXO9 overexpression using a histidine-based ubiquitination assay (Supplementary Fig. S4B). This suggested the presence of potential ubiquitination sites between lysine residue 132 and 414 (Supplementary Fig. S4B). Subsequently, we identified eight specific lysine residues that appeared to be crucial for ATP6V1A ubiquitination. Remarkably, reintroducing lysine residue 393 (K393) into ATP6V1A-KR significantly enhanced ubiquitination signals according to a histidine-based ubiquitination assay (Fig. 4A and Supplementary Fig. S4C). This finding emphasizes the vital role of K393 in FBXO9-mediated ATP6V1A ubiquitination. Interestingly, substituting K393 with arginine in wild-type ATP6V1A to generate the K393R mutant nearly abolished ATP6V1A ubiquitination (Fig. 4B). Furthermore, cross-species analysis demonstrated the conservation of K393 (Fig. 4C). The K393R mutation in Atp6v1a significantly decreased Atp6v1a ubiquitination compared to wild-type Atp6v1a when Fbxo9 was overexpressed (Fig. 4D). This confirmed the ubiquitination site.

We established H1299 cell lines expressing sgRNA-resistant ATP6V1A in either its wild-type or K393R mutant form using a Tet-Off expression system to further explore the biological implications of the ATP6V1A ubiquitin modification at K393. We selectively depleted endogenous ATP6V1A and reconstituted the V-ATPase complex using CRISPR. Immunoblot analysis confirmed that the ectopic expression levels of ATP6V1A were comparable to those in the control cells and the presence of endogenous subunits such as ATP6V1B and ATP6V1D1 was unaffected by ATP6V1A expression (Fig. 4E). These findings indicate that reconstitution of the V-ATPase complex through ectopic expression of ATP6V1A accurately mimics physiological conditions. Transwell migration and tumor sphere formation assays demonstrated that the ATP6V1A-K393R mutant significantly enhanced cell migration (Fig. 4F and G) and tumor sphere growth (Fig. 4H and I), which is consistent with observations of previous studies showing the augmented effects of FBXO9 depletion (Fig. 1H-K). Furthermore, knockdown of ATP6V1A in cancer cell lines using shRNA inhibited cancer cell migration in mouse and human cell models (Supplementary Fig. S5A-F). These findings suggested a potential connection between FBXO9-mediated ATP6V1A ubiquitination and its inhibitory regulatory effect on the protein.

To gain further insights, we generated cancer cell lines with inducible expression of V1A-23 aa (a peptide that inhibits ATP6V1A ubiquitination) by disrupting the interaction between FBXO9 and ATP6V1A (Supplementary Fig. S6A-D). Cell migration assays and tumor sphere formation experiments revealed that the expression of V1A-23 aa significantly enhanced tumor cell migration (Fig. 4J-L and Supplementary Fig. S7A and B) and tumor sphere formation (Fig. 4M and N, Supplementary Fig. S7C and D) in both the A549 and H1299 cell lines. These data support previous results suggesting that the FBXO9 inhibition enhances the migration phenotype of cancer cells (Fig. 1H-K). We injected H1299 cells expressing V1A-23 aa into the mouse tail vein to assess the effect of FBXO9-mediated inhibition of ATP6V1A ubiquitination on lung metastasis in vivo. Microscopic analysis and hematoxylin-eosin (H&E) staining of mouse lungs at six weeks post-inoculation demonstrated a substantial increase in lung metastatic nodules in the group expressing V1A-23 aa compared with that in the control samples (Fig. 4O and P). This effect was similar to that observed when depleting FBXO9 in the H1299 cell metastasis mouse model. Hence, these in vivo results confirm the potential role of FBXO9 as an inhibitory protein against the migration, tumor sphere growth, and metastasis of lung cancer cells by promoting ATP6V1A ubiquitination.

### ATP6V1A ubiquitination mediated by FBXO9 decreases V-ATPase assembly

**Fig. 5:**
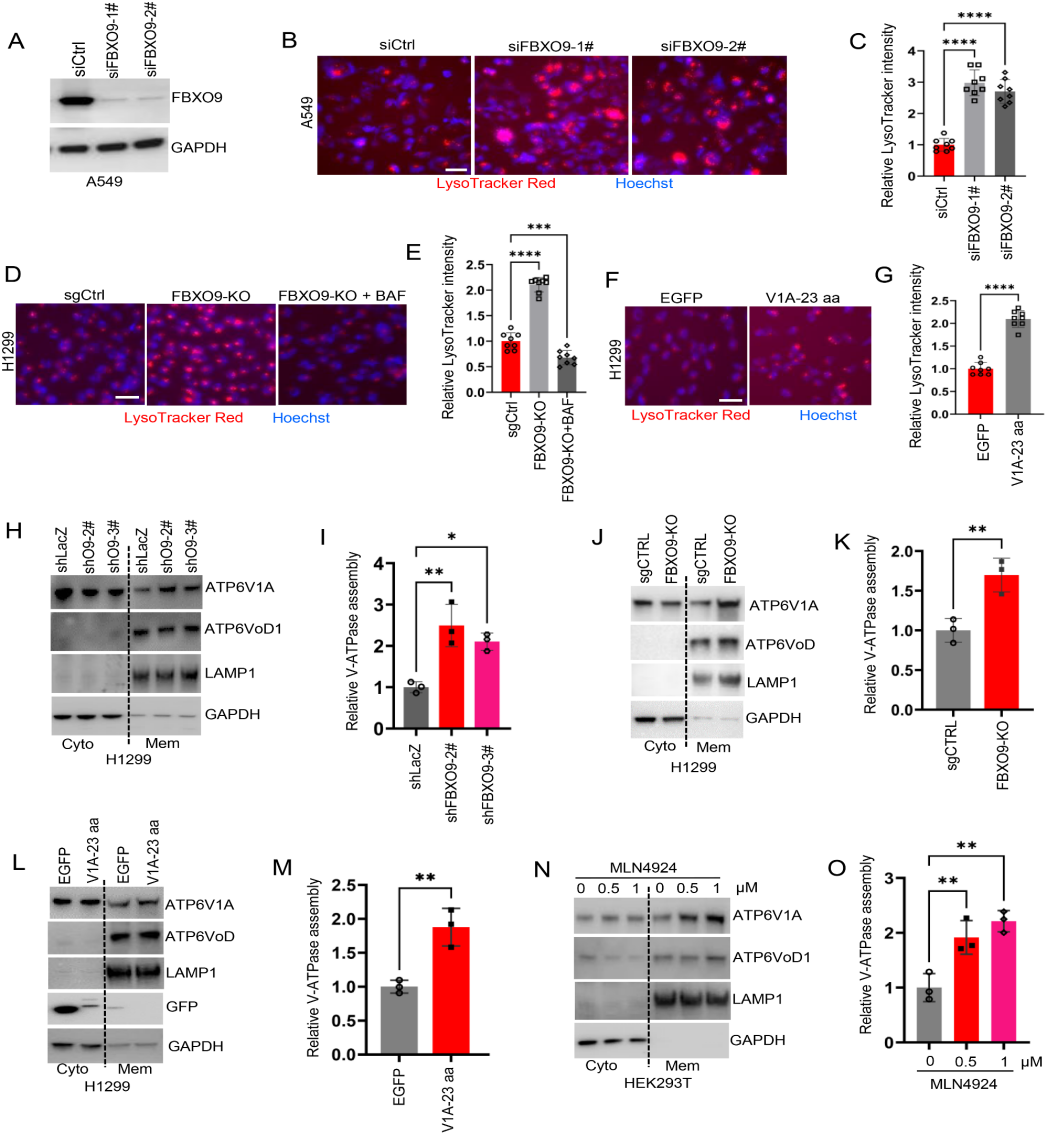
ATP6V1A ubiquitination by FBXO9 impairs V-ATPase assembly. **A**–**C** Assessment of lysosomal acidity in A549 cells after FBXO9 knockdown. A549 cells were transfected with FBXO9 siRNA and stained with LysoTracker Red. Plots show the overall LysoTracker intensity per cell (Scale bar = 100 μm, *n* = 6**–**8 images from three independent experiments, ****P < 0.0001). **D, E** Representative images demonstrate LysoTracker Red staining in negative control and FBXO9-knockout H1299 cells. FBXO9-knockout H1299 cells treated with 200 nM bafilomycin A1 (BAF) for 12 h were used as a rescue control. The average staining intensity was quantified as in C. Scale bar = 100 μm, ***P < 0.0001 and ****P < 0.0001. **F, G** Representative images demonstrate LysoTracker Red staining in H1299 cells ectopically expressing V1A-23 aa. The average staining intensity was quantified using the method described in C. **H, I** Analysis of V-ATPase assembly in FBXO9-knockdown H1299 cells. FBXO9 was depleted using shRNA, followed by subcellular fractionation. Immunoblot analysis was conducted using antibodies targeting ATP6V1A and ATP6VoD (H). LAMP1 and GAPDH were used as loading controls for membrane and cytosolic proteins, respectively. Ratio of ATP6V1A (*n* = 3 independent experiments, *P < 0.05, **P < 0. 01) to ATP6VoD in the membrane fraction represents the assembly of the V-ATPase (I). **J, K** Analysis of V-ATPase assembly in FBXO9-knockout H1299 cells. sgCtrl and FBXO9-knockout H1299 cells were fractionated, followed by immunoblot analysis of ATP6V1A and ATP6VoD (J). Levels of assembled ATP6V1A (K) (*n* = 3 independent experiments, **P < 0.01) were normalized against ATP6VoD in the membrane fraction. **L, M** Analysis of V-ATPase assembly in H1299 cells ectopically expressing V1A-23 aa or EGFP is depicted. Representative immunoblots for ATP6V1A, ATP6VoD, LAMP1, and GAPDH are presented (L). The levels of assembled ATP6V1A (M) (*n* = 3 independent experiments, **P < 0. 01) are measured plotted, and normalized against ATP6VoD in the membrane fraction (M). **N, O** HEK293T cells were treated with MLN4924 at indicated concentrations for 12 h. Immunoblotting using antibodies against ATP6V1A and ATP6VoD was performed on cytosolic and membrane fractions (N). The levels of assembled ATP6V1A (O) (*n* = 3 independent experiments, **P < 0. 01) were determined as described in panel (M)

ATP6V1A plays a crucial role as a component of the catalytic head group of the V-ATPase V1 domain and significantly influences lysosomal acidification [9]. We initially reduced FBXO9 expression in A549 lung cancer cells by siRNA transfection to investigate the effect of ATP6V1A ubiquitination on V-ATPase (Fig. 5A). The effect on lysosomal pH was evaluated using LysoTracker Red, a specialized red fluorescent probe that specifically accumulates in acidic organelles such as lysosomes. Notably, we observed a significant increase in red fluorescence intensity within cells upon FBXO9 knockdown (Fig. 5B and C). Similar increases in fluorescence intensity were observed using the FBXO9-knockout H1299 cell line as an additional model to further support these findings. Notably, treatment with BAF (a specific inhibitor of V-ATPase) significantly decreased the red fluorescence intensity in FBXO9-knockout H1299 cells (Fig. 5D and E). This indicated a strong connection between FBXO9 and V-ATPase-regulated lysosomal acidity. Based on this observation, we aimed to alleviate intracellular ATP6V1A ubiquitination in H1299 cells by introducing V1A-23 aa via ectopic expression. This intervention resulted in a notable increase in red fluorescence intensity (Fig. 5F and G), which suggested that FBXO9-driven ATP6V1A ubiquitination impairs V-ATPase complex activity.

Disassembly and assembly of the V1 and Vo domains of V-ATPase on the vesicular membrane are the primary mechanisms regulating V-ATPase activity [10, 14]. We examined the levels of the assembled V-ATPase V1-Vo holoenzymes on the vesicular membrane to understand how FBXO9-mediated ATP6V1A ubiquitination affects the activity of the V-ATPase complex. This analysis was performed using FBXO9 knockdown or knockout cells. We isolated the cell membrane fraction and quantified the abundance of membrane-associated V1A subunit relative to that of the VoD subunit. This served as an indicator of V1-Vo holoenzyme assembly. Our findings demonstrated a significant increase in the docking level between the V1 and Vo domains in FBXO9-knockdown H1299 cells using shRNA and FBXO9-knockout H1299 cells (Fig. 5H-K). This increase was supported by the presence of ATP6V1A in the membrane components. Additionally, we observed a parallel promotive effect of Fbxo9 knockout on increased vesicular acidification and ATP6V1A localization to the membrane of mouse-derived 889DTC cells (Supplementary Fig. S8A and B). Overall, these findings suggest that the downregulation of FBXO9 expression facilitates V-ATPase assembly, resulting in improved lysosomal acidification.

We blocked ATP6V1A ubiquitination in H1299 cells by introducing V1A-23 aa to investigate the specific role of FBXO9-mediated ATP6V1A ubiquitination in V-ATPase assembly. Interestingly, this approach significantly enhanced the membrane localization of ATP6V1A, which is similar to that observed in FBXO9-depleted cells (Fig. 5L and M). Furthermore, our study demonstrated that neddylation inhibition by MLN4924 promoted the membrane localization of ATP6V1A (Fig. 5N and O). These collective observations provide further evidence that FBXO9-mediated ATP6V1A ubiquitination negatively regulates the activity of the V-ATPase complex by obstructing the assembly of V-ATPase V1-Vo holoenzymes in lysosomal membranes.

### HSPA8-mediated cytoplasmic sequestration of ubiquitinated ATP6V1A by FBXO9

**Fig. 6:**
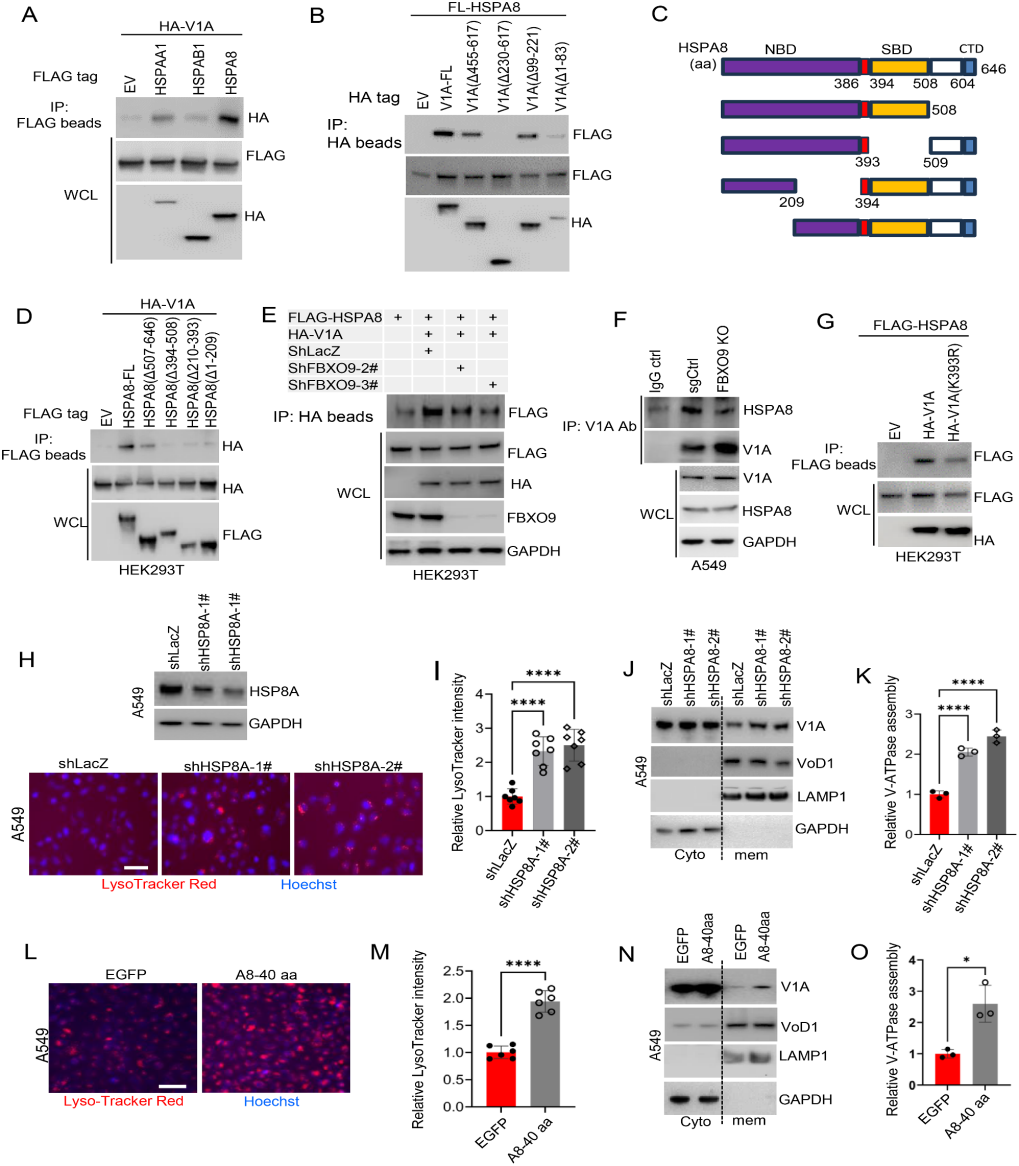
FBXO9-mediated ATP6V1A ubiquitination leading to cytoplasmic localization facilitated by HSPA8 **A** Interaction between HSPA8 and ATP6V1A assessed by co-IP assay. HEK293T cells were co-transfected with indicated constructs. After 36 h, co-IP and immunoblot was performed to detect the associated HA-ATP6V1A using anti-HA antibodies. **B** Mapping the binding domain of ATP6V1A with HSPA8. Different versions of HA-tagged ATP6V1A, including full-length (FL) and truncated constructs, were co-transfected with FLAG-HSPA8 in HEK293T cells. Co-IP was performed to assess the interaction, and the presence of bound FLAG-HSPA8 was detected using anti-FLAG antibodies. **C, D** Mapping the binding domain of HSPA8 with ATP6V1A. Different forms of FLAG-tagged HSPA8 constructs, including full-length (FL) and truncated versions, were designed and shown (C). HEK293T cells were co-transfected with HA-ATP6V1A and the corresponding FLAG-tagged HSPA8 constructs. Co-IP assay was performed using anti-FLAG beads to assess the interaction between HSPA8 and ATP6V1A. The presence of bound HA-ATP6V1A was detected using anti-HA antibodies (D). **E** Impact of FBXO9 knockdown on the interaction between ATP6V1A and HSPA8. HEK293T cells pre-treated with FBXO9-shRNA were co-transfected with HA-ATP6V1A and FLAG-HSPA8. After 36 h, a co-IP assay was performed to evaluate the effect of FBXO9 depletion on the interaction between ATP6V1A and HSPA8. **F** Effect of FBXO9 knockout on the interaction between endogenous ATP6V1A and HSPA8. FBXO9-knockout H1299 was used and a co-IP assay was performed using anti-ATP6V1A antibody. The presence of the bound endogenous HSPA8 was detected using anti-HSPA8 antibodies. **G** Role of FBXO9-mediated ubiquitination in modulating the interaction between ATP6V1A and HSPA8. HEK293T cells were co-transfected with FLAG-HSPA8 and either HA-ATP6V1A or HA-ATP6V1A-K393R. The co-IP assay was then conducted to evaluate the interaction between HSPA8 and ATP6V1A. **H, I** Impact of HSPA8 depletion on lysosomal acidity in A549 cells. HSPA8 was knocked down using shRNA (H, upper). The cells were stained with LysoTracker Red and fluorescence was measured (H, lower). Staining intensity was quantified as outlined in Fig. 5C (I). The scale bar is 50 µM; (*P* < 0.0001). **J, K** Impact of HSPA8 depletion on V-ATPase assembly in A549 cells. HSPA8-knockdown cells were fractionated, and immunoblotting was performed using antibodies against ATP6V1A and ATP6VoD (J). Membrane and cytosolic proteins were assessed with LAMP1 and GAPDH as loading controls, respectively. Levels of assembled ATP6V1A were measured and normalized to ATP6VoD in the membrane fraction (K). Results are from three independent experiments (*P* < 0.0001). **L, M** Blocking the interaction between HSPA8 and ATP6V1A enhances lysosomal acidity in A549 cells. Cells expressing the A8-40 aa peptide were stained with LysoTracker Red and fluorescence was observed (L). The average staining intensity was quantified as outlined in Fig. 5C. Scale bar = 100 µM. ****P < 0.0001. **N, O** Blocking the interaction between HSPA8 and ATP6V1A promotes V-ATPase assembly. A549 cells expressing the A8-40 aa peptide were fractionated. Immunoblot analysis was then performed to assess the level of V-ATPase in the cells (*P* < 0.05), as described in Fig. 6J and K

We further examined how the downregulation of FBXO9 expression aids in the assembly of the V-ATPase holoenzyme on the vesicular membrane. We hypothesized that the underlying mechanisms could be clarified by investigating the proteins that interact with ATP6V1A. Thus, we directed our empirical screening to the cytoplasmic chaperones HSP90AA1, HSP90AB1, and HSPA8, which are associated with ATP6V1A (Fig. 2B). We individually transfected ATP6V1A constructs with HSP90AA1, HSP90AB1, and HSPA8 into HEK293T cells and conducted an IP assay. The results indicated substantial co-IP of HSPA8 with ATP6V1A, whereas the remaining two chaperones showed minimal binding affinity for HSPA8 (Fig. 6A). We then performed domain-mapping experiments and reciprocal IP assays using truncated versions of epitope-tagged HSPA8 and ATP6V1A to investigate the interaction between HSPA8 and ATP6V1A. Immunoblot analysis revealed that the amino acid regions between 1 and 83 aa and 230–617 aa in ATP6V1A were crucial for its interaction with HSPA8 (Fig. 6B). Additionally, a similar strategy confirmed that either the substrate-binding domain (SBD) or the nucleotide-binding domain of HSPA8 was necessary for its interaction with ATP6V1A (Fig. 6C and D). These findings establish a strong correlation between HSPA8 and ATP6V1A expression.

shRNA was used to downregulate FBXO9 expression in HEK293T cells to explore the effect of FBXO9 on the interaction between HSPA8 and ATP6V1A. Subsequently, we co-transfected HSPA8 and ATP6V1A and then performed IP assay to assess their interactions. Immunoblot analysis revealed a decrease in the binding affinity of HSPA8 for ATP6V1A upon FBXO9 depletion (Fig. 6E). Furthermore, we used an ATP6V1A-specific antibody to immunoprecipitate ATP6V1A and detect co-immunoprecipitated endogenous HSPA8 in FBXO9-knockout H1299 cells. FBXO9 depletion significantly inhibited the interaction between ATP6V1A and HSPA8 (Fig. 6F), which indicates that FBXO9 has a substantial role in facilitating this interaction. We then transfected HSPA8 with wild-type ATP6V1A or ATP6V1A-K393R in HEK293T cells to further support this hypothesis. The IP assay confirmed that the ATP6V1A-K393R mutant exhibited a diminished binding capacity towards HSPA8 compared with that of the wild-type form (Fig. 6G). Collectively, these results established that FBXO9-mediated ubiquitination of ATP6V1A enhances its interaction with HSPA8 in the cellular environment.

We investigated the functional implications of the interaction between ATP6V1A and HSPA8 by inhibiting HSPA8 expression in A549 cells using shRNA and examining the effect of HSPA8 depletion on lysosomal pH changes using LysoTracker Red. A significant increase in red fluorescence intensity was observed within the cells upon reduction in HSPA8 levels (Fig. 6H and I). This observation strongly indicated that HSPA8 plays a crucial role in regulating lysosomal acidification, which is primarily controlled by V-ATPase. We performed shRNA-mediated knockdown of HSPA8 in A549 cells to evaluate V-ATPase assembly at the vesicular membrane to understand the mechanism underlying this regulation. Remarkably, A significant increase in the relative abundance of the V1 domain docked with the Vo domain was observed when HSPA8 was reduced compared to that in the control cells (Fig. 6J and K). These findings strongly support the idea that HSPA8 negatively regulates V-ATPase assembly within cells. Based on the observed interaction between HSPA8 and ATP6V1A, we hypothesized that HSPA8 sequesters ATP6V1A in the cytosol to hinder its localization to the vesicular membrane. This was investigated by designing constructs producing peptides of varying lengths (HSPA8-25 aa, 40 aa, 50 aa, and 60 aa) that target the SBD of the HSPA8 protein (Supplementary Fig. S9A). IP assays confirmed that most of these HSPA8 peptides (except for HSPA8-60 aa) effectively disrupted the ATP6V1A and HSPA8 interactions in the cellular environment (Supplementary Fig. S9B). Subsequently, HSPA8-40 aa was selected, and an A549 cell line with ectopic expression of this peptide was established. LysoTracker Red staining and subcellular fractionation revealed a significant increase in red fluorescence intensity (Fig. 6L and M) and ATP6V1A protein localization to the vesicular membrane (Fig. 6N and O). These findings strongly support the hypothesis that FBXO9-mediated ATP6V1A ubiquitination facilitates its interaction with the chaperone protein HSPA8, thus leading to cytosolic sequestration and V-ATPase assembly inhibition.

### FBXO9 inhibits wnt signaling and epithelial-mesenchymal transition by ubiquitinating ATP6V1A

**Fig. 7:**
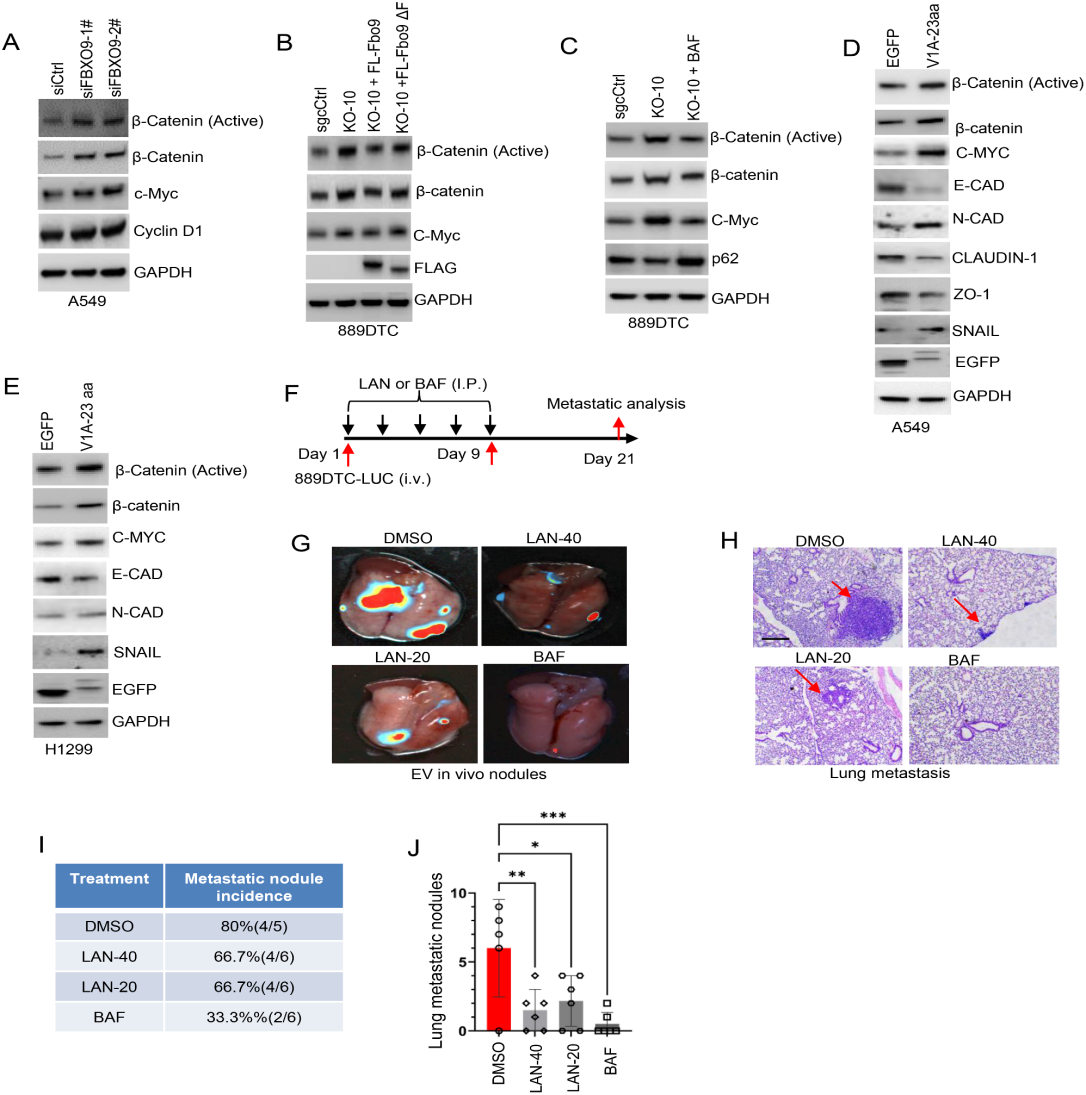
ATP6V1A ubiquitination by FBXO9 suppresses Wnt signaling and EMT **A** Immunoblot analysis of active β-catenin (non-phospho-β-Catenin in Ser45) levels and other indicated proteins in A549 cells transfected with siCtrl or siFBXO9. GAPDH was used as a loading control throughout. **B** Immunoblot analysis showing the levels of active β-catenin (non-phospho-β-catenin in Ser45) and indicated proteins in 889DTC cells. The cells were either Fbxo9-knockout or Fbxo9-knockout cells reintroduced with full-length Fbxo9 or Fbxo9-ΔF. **C** Immunoblot analysis illustrating the levels of active β-catenin and indicated proteins in 889DTC cells with Fbxo9-knockout or Fbxo9-knockout 889DTC cells treated with 200 nM bafilomycin A1 (BAF) for 12 h. **D, E** Immunoblot analysis illustrating the expression levels of active β-catenin and EMT markers in A549 (d) or H1299 (e) cells ectopically expressing V1A-23 aa. **F** Experimental setup for tail vein lung metastasis treatment. Nude mice were injected with 1 × 10^5^ 889DTC-LUC cells by vein tail route and received intraperitoneal treatment with a vehicle (5% DMSO in corn oil), BAF (1 mg/kg), lansoprazole (LAN) at 20 mg/kg, or LAN at 40 mg/kg (*n* = 5–6 mice per group). The treatments were administered every other day for a total of five consecutive treatments over a 21-day period. **G, H** Lungs of mice were collected on day 21 for both bioluminescent imaging (G) and histological examination using H&E staining (H). The arrows in the image point towards the location of the tumor within the lungs. Scale bar, 100 μm. **I** Table displaying the incidence of metastatic nodules following different treatments. **J** Quantitative evaluation of lung metastatic nodules. Data are expressed as the mean ± SD. *P < 0.05, **P < 0.01, ***P < 0.001

V-ATPase is a crucial enzyme that plays a significant role in initiating cancer signaling pathways (including Wnt and Notch) by regulating vesicular acidification [18, 19]. The Wnt/β-catenin pathway is particularly important for cancer development [46]. Our investigation aimed to understand the impact of FBXO9 on the Wnt/β-catenin pathway in lung cancer cells. Silencing FBXO9 in A549 cancer cells by siRNA transfection substantially increased the expression of active β-catenin (the primary effector of the canonical Wnt signaling pathway), C-Myc and cyclin D1 (both important downstream targets of this pathway) upon FBXO9 depletion (Fig. 7A). Fbxo9-knockout 889DTC cells showed elevated levels of active β-catenin, C-Myc, and cyclin D1 (Fig. 7B). This was consistent with the results observed in FBXO9-knockdown A549 cells. Remarkably, reintroducing the entire length of Fbxo9 into these Fbxo9-deficient cells restored decreased levels of active β-catenin, C-Myc, and cyclinD1. However, reintroduction of the Fbxo9-ΔF mutant failed to produce the same effect (Fig. 7B). These findings suggest a negative correlation between FBXO9 expression and Wnt/β-catenin signaling activation, which is dependent on the ligase activity of FBXO9. As previous experiments showed that FBXO9 suppresses V-ATPase through ATP6V1A ubiquitination, we sought to explore the relationship between FBXO9 and V-ATPase in the regulation of Wnt/β-catenin signaling in lung cancer cells (Fig. 5). Therefore, Fbxo9-knockout 889DTC cells were treated with BAF. The inhibition of V-ATPase by the specific inhibitor led to a significant reduction in the expression of active β-catenin, regardless of the absence of FBXO9 (Fig. 7C). These data highlight the compelling role of V-ATPase activity in the regulatory function of FBXO9 in the Wnt/β-catenin signaling pathway.

We utilized A549 and H1299 cell lines expressing V1A-23 aa to block ATP6V1A ubiquitination to investigate the specific effect of FBXO9- mediated ATP6V1A ubiquitination on Wnt/β-catenin signaling. An increase in active β-catenin expression was observed when ATP6V1A ubiquitination was inhibited using this peptide (Fig. 7D and E). These findings were consistent with those observed in FBXO9-depleted cells (Fig. 7A and B). We further explored the impact of ATP6V1A ubiquitination on epithelial-mesenchymal transition (EMT) in cancer cells since the Wnt/β-catenin signaling pathway plays a role in regulating transcriptional changes during EMT [47]. Immunoblot analysis revealed that inhibiting ATP6V1A ubiquitination through the expression of V1A-23 aa promotes EMT, which was supported by the downregulation of the epithelial marker E-cadherin and alterations in other EMT markers (Fig. 7D and E). Collectively, these findings provide substantial evidence supporting the significant role of FBXO9-mediated ATP6V1A ubiquitination (which hinders V-ATPase assembly) in suppressing Wnt/β-catenin signaling and EMT in lung cancer cells.

We hypothesized that a direct blockade of V-ATPase could effectively mitigate lung cancer metastasis after confirming the inhibitory effect of FBXO9 on the Wnt/β-catenin signaling pathway through targeted V-ATPase inhibition. This was investigated using BAF and LAN proton pump inhibitors, which can also inhibit V-ATPase activity [48]. Our initial experiments showed that LAN exhibited varying degrees of inhibition on cell migration and clonogenic survival (Supplementary Fig. S10A-D). Furthermore, we utilized FBXO9-knockdown H1299 and Fbxo9-knockout 889DTC cells as models and validated the efficacy of LAN in inhibiting V-ATPase activity to highlight its potential as an anti-metastatic agent (Supplementary Fig. S10E-H). We administered lateral tail vein injections of 889DTC-Luciferase cells in a mouse model to evaluate the efficacy of BAF and LAN in inhibiting lung metastasis in vivo (Fig. 7F). After three weeks of treatment, the mice were euthanized and their lungs were collected for analysis. Bioluminescent imaging revealed a significantly reduced tumor burden in the lungs of mice treated with BAF (1 mg/kg) or LAN (40 and 20 mg/kg) compared with that of the control group (Fig. 7G). These results are consistent with the presence of fewer metastatic lesions observed by H&E staining (Fig. 7H). Notably, BAF exhibited a more pronounced inhibitory effect on metastasis than LAN (Fig. 7I and J). These findings suggest that targeting V-ATPase under FBXO9 regulation is a promising strategy against lung cancer metastasis.

### FBXO9 expression indicates lung cancer patient outcomes

**Fig. 8:**
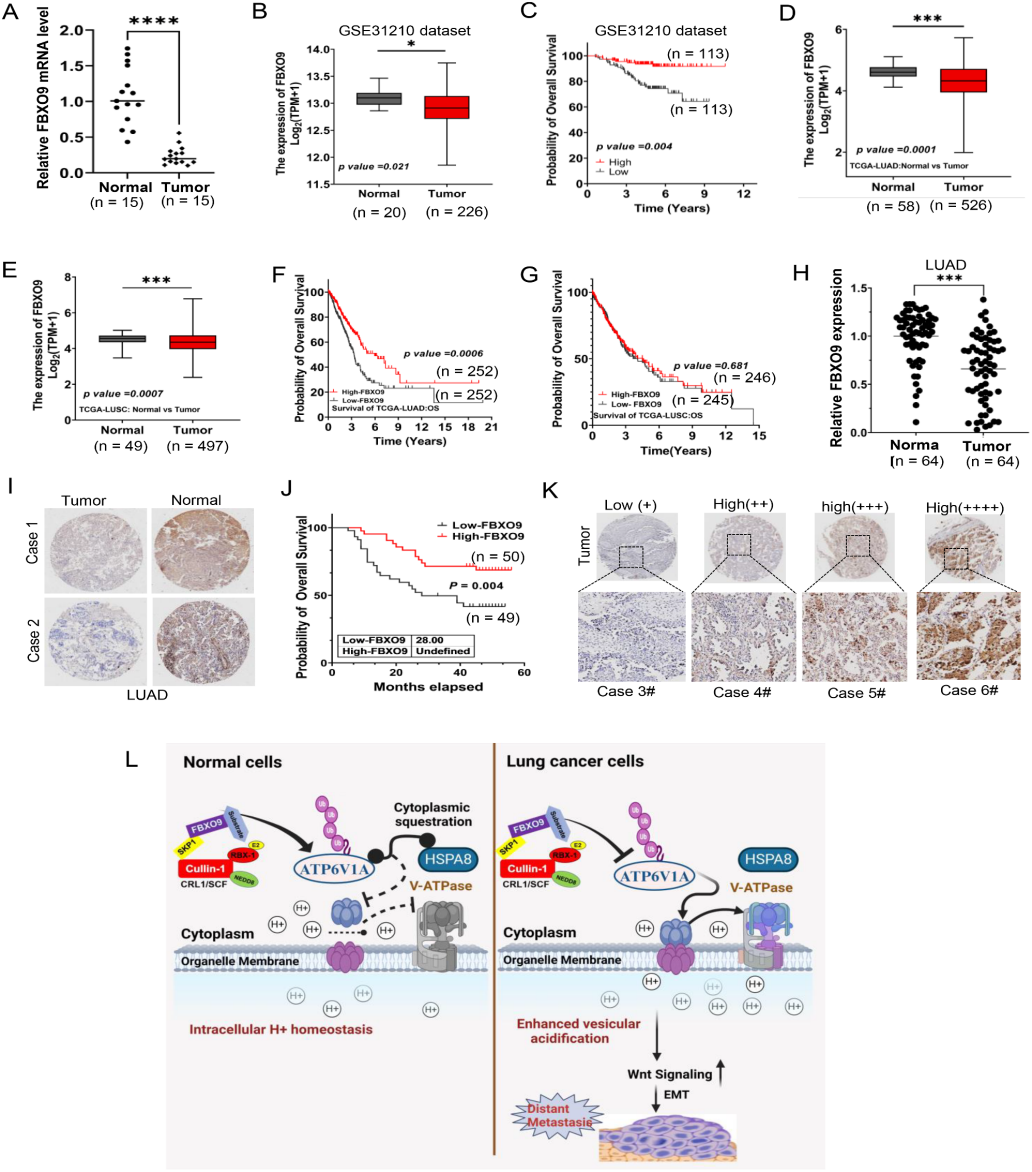
Expression of FBXO9 in lung cancer tissues and their association with patient survival **A** Comparison of FBXO9 mRNA expression between tumor and normal tissues by quantitative PCR using cDNA microarray (*P* < 0.0001). The bar graph shows significantly lower FBXO9 mRNA expression in tumor tissues (*n* = 15) compared to normal tissues (*n* = 15). **B** Analysis of GSE31210 dataset revealed a significant decrease in FBXO9 mRNA expression in lung cancer tissues (*n* = 113) compared to normal control tissues (*n* = 113) (*P* = 0.021). **C** Kaplan–Meier survival analysis of FBXO9 expression from GSE31210 dataset demonstrated that a higher level of FBXO9 mRNA was associated with a better overall survival (*P* = 0.004). **D, E** FBXO9 mRNA expression in lung cancer. Data analysis from TCGA database reveals that the mRNA expression of FBXO9 is notably downregulated in both LUAD (*P* = 0.0001) (D) and LUSC (*P* = 0.0007) (E) tissues when compared to their corresponding normal control tissues. **F, G** Prognostic value of FBXO9 mRNA expression in Lung Cancer. Data analysis from the TCGA database indicates that relatively lower expression of FBXO9 is associated with worse overall survival (OS) in lung cancer (*P* = 0.0006) (F). However, there is no significant difference in OS between patients with high and low expression of FBXO9 (*P* = 0.681) (G). **H, I** Protein expression of FBXO9 in LUAD. Immunohistochemistry (IHC) analysis on TAM samples (H) shows significantly lower FBXO9 levels in LUAD tissues compared to adjacent normal tissues (*P* < 0.001) (I). **J** Kaplan–Meier survival analysis of FBXO9 expression in TMA samples demonstrated that higher levels of FBXO9 protein correlated with improved overall survival (*P* = 0.004). **K** Representative images depicting the differential expression of FBXO9 protein in TMA samples. **L** FBXO9 controls lung cancer metastasis. A working model is as follows: In normal cells, FBXO9 promotes the ubiquitination of ATP6V1A, which helps maintain balanced cellular acidity by sequestering ATP6V1A in the cytoplasm and hindering V-ATPase assembly. However, in lung cancer cells, reduced FBXO9 levels disrupt ATP6V1A ubiquitination, leading to increased V-ATPase assembly and enhanced acidification of vesicles. The excessive acidification activates the Wnt signaling pathway and EMT, thereby driving the metastasis of lung cancer cells to distant sites. *P < 0.05, **P < 0.01, ***P < 0.001

We performed quantitative PCR using cDNA microarrays to compare the levels of FBXO9 mRNA in 15 LUAD specimens and their respective non-cancerous tissues to ascertain the clinical significance of our findings. Our analysis revealed a significant decrease in FBXO9 mRNA expression in tumor tissues compared to non-cancerous tissues (*P* < 0.0001; Fig. 8A). We analyzed the GSE31210 dataset from the GEO database, which included the mRNA expression profiles of 226 patients with lung cancer. The patients were divided into two groups based on their FBXO9 mRNA expression levels. Dataset analysis confirmed a significant reduction in FBXO9 mRNA expression in lung cancer cases (*P* = 0.021; Fig. 8B). This was consistent with the data presented in Fig. 8A. Furthermore, elevated survival rates were observed among patients with augmented levels of FBXO9 mRNA compared to those with diminished levels (*P* = 0.004; Fig. 8C). An additional examination was conducted using TCGA database to bolster these findings; it confirmed that FBXO9 mRNA expression was significantly downregulated in patients with LUAD and LUSC (*P* < 0.0001; Fig. 8D and E) and revealed improved survival rates among LUAD patients with higher levels of FBXO9 mRNA (*P* = 0.0006; Fig. 8F). However, no substantial association was observed between FBXO9 mRNA expression and survival rates in patients with LUSC (*P* = 0.681; Fig. 8G). These findings highlighted the clinical significance of decreased FBXO9 mRNA expression in LUAD.

To further substantiate our findings, we performed immunohistochemical analysis of tumor tissue microarray (TMA) samples collected from patients with LUAD. Immunohistochemistry analysis confirmed reduced FBXO9 expression in tumor tissues compared with that in adjacent normal tissues (*P* < 0.0001; Fig. 8H and I), which was consistent with our mRNA expression data. We analyzed a unique set of TMA samples from a different group of patients with LUAD enriched with overall patient survival data to strengthen our results and examine the relationship between FBXO9 expression and survival. A significant association was observed between decreased FBXO9 expression and adverse survival outcomes in patients with lung cancer (*P* = 0.004; Fig. 8J and K). This suggested that FBXO9 represents a potential prognostic factor for lung cancer. Overall, our comprehensive analyses of mRNA and protein levels consistently demonstrated that FBXO9 is significantly downregulated in lung cancer. The correlation between decreased FBXO9 expression and unfavorable survival outcomes further emphasized the clinical importance of our findings, thus highlighting FBXO9 as a promising prognostic marker and a potential therapeutic target for lung cancer.

## Discussion

This study explored the functional role of FBXO9 in lung cancer. The biological activities of FBXO9 including inhibition of lung cancer cell migration, retardation of tumor sphere growth, and restriction of metastasis, were demonstrated in a preclinical setting. Moreover, a comprehensive analysis was conducted to unravel the tripartite interplay between FBXO9, the cytoplasmic chaperone HSPA8, and the ATP6V1A subunit of the V-ATPase complex. These findings provide compelling evidence that FBXO9 impedes the assembly and activity of the V-ATPase complex by promoting non-degradative ubiquitination of ATP6V1A, which is sequestered in the cytosol by HSPA8. This study underscored the crucial role of FBXO9 in controlling V-ATPase assembly and proposed a molecular blueprint to clarify the role of FBXO9 in combating lung cancer cell metastasis.

The mechanisms underlying the effects of FBXO9 on cancer progression are poorly understood. Previous studies have shown that FBXO9 expression is reduced in the bone marrow of patients with acute leukemia [36]. This observation was confirmed in a mouse model, wherein the lack of FBXO9 accelerated the progression of acute myeloid leukemia and increased proteasome activity [36]. Although these studies focused on different mechanisms, they were aligned with our understanding of the anticancer effects of FBXO9 based on a lung cancer model. Our study proposes that FBXO9 plays a role in mitigating lung cancer metastasis, which is supported by several important observations. First, overexpression of FBXO9 in lung cancer cells reduced the migration of these cells in vitro. Additionally, it inhibits the formation of tumor spheres and prevents the emergence of metastatic sites in the lungs via a tail vein injection model using 889DTC cells (*Kras*^G12D^/*Trp53*^−/−^). In contrast, downregulation of FBXO9 had the opposite effect on the cancer phenotypes, promoting lung cancer metastasis in H1299 cancer cells employing a similar mouse model. Furthermore, FBXO9 expression was low in lung cancer tissues and inversely correlated with the survival rates of patients with lung cancer. This suggests that FBXO9 could potentially serve as a prognostic biomarker for lung cancer; however, further validation with additional samples is required.

Compared with its role in lung cancer, FBXO9 presents higher expression levels and oncogenic activity in other conditions, such as multiple myeloma [35] and hepatocellular carcinoma [34]. FBXO9 promotes the ubiquitination and degradation of the mTOR activators TEL2 and TTI1 [35] and tumor suppressor FBXW7 [34], which is another member of the F-box protein family. The specific biological functions of FBXO9 may vary across different types of tumors and are potentially influenced by organ- or tissue-specific differences. Figure 1 depicts the correlation between FBXO9’s suppressive effect on lung cancer cells and its ubiquitin ligase activity. Therefore, we focused on the suppressive effect of FBXO9 on lung cancer cells via its ubiquitin ligase activity to better understand how FBXO9 controls lung cancer. Through meticulous biochemical analyses, we have identified ATP6V1A as a novel substrate of FBXO9. This discovery is significant because V-ATPase dysfunction has been linked to tumor progression. Importantly, the direct inhibition of FBXO9-mediated ATP6V1A ubiquitination by an interaction inhibitory peptide not only prevents cancer cell migration and sphere formation (Fig. 4H-N) but also hinders the metastasis of lung cancer cells in a mouse model (Fig. 4O and P). These findings collectively establish the causal role of ATP6V1A ubiquitination by FBXO9 in inhibiting lung cancer metastasis.

The activity of the V-ATPase complex that is responsible for maintaining pH balance within cellular compartments, is primarily regulated by its reversible disassembly and assembly [13, 14]. However, the signaling pathways that control these processes are poorly understood. This study provides new evidence highlighting the role of FBXO9 in inhibiting the assembly of the V-ATPase complex through increased non-degradative ubiquitination of ATP6V1A. This discovery raises questions about how the ubiquitination of ATP6V1A affects the assembly of the V-ATPase complex. Our results showed that ATP6V1A specifically interacted with the cytosolic chaperone HSPA8 following FBXO9-mediated ubiquitination, which resulted in HSPA8 sequestering ATP6V1A to prevent its integration into the V-ATPase complex. Consequently, the proper assembly of the complex was impeded, thus leading to compromised vesicular acidification (Fig. 8L). These findings provide valuable insights into the regulatory mechanisms governing V-ATPase complex assembly. They also shed light on the complex interplay between ubiquitin ligases (such as FBXO9) and chaperones (such as HSPA8) in modulating this crucial cellular process. Further understanding of these mechanisms may have implications for the development of new therapeutic strategies targeting the V-ATPase complex in diseases such as cancer where the dysregulation of pH homeostasis plays a role in progression and metastasis.

V-ATPases play critical roles in various physiological functions. However, they are often overexpressed or hyperactive under tumor conditions, thus leading to tumor cell proliferation, survival, spread, and resistance to treatment [18, 19, 21]. V-ATPases regulate intracellular tumor signaling pathways such as Wnt, Notch, and mTOR [19, 21]. Recent studies have linked V-ATPase-induced activation of the Wnt signaling pathway to intracellular acidification, which is associated with the development and progression of colorectal and liver cancers [49, 50]. Our results suggest that knocking out FBXO9 or inhibiting ATP6V1A ubiquitination increases V-ATPase assembly and activity, which is consistent with previous studies. Such changes increase intracellular Wnt signaling, as indicated by the upregulation of active β-catenin (Fig. 7A-E). This insight enhances our understanding of the potential pathological implications of abnormally low FBXO9 expression in tumor tissues because it may lead to uncontrolled activation of Wnt signaling. Additionally, we propose that the downregulation of FBXO9 expression or inhibition of ATP6V1A ubiquitination accelerates EMT. Recent studies have suggested that the proton pump inhibitor omeprazole potentially inhibits EMT in tumor cells by resolving intracellular acidification and promoting the epithelial marker protein E-cadherin degradation [51]. Despite the direct involvement of the Wnt signaling pathway in promoting EMT, whether FBXO9-regulated EMT is facilitated by the inhibition of E-cadherin degradation remains unclear. Further research is needed to investigate the precise mechanisms by which FBXO9 regulates EMT and the potential therapeutic implications of targeting FBXO9 and V-ATPase activity in tumor progression and metastasis.

We have determined that the reduced expression of FBXO9 in lung cancer cells leads to intracellular acidification, which in turn activates Wnt signaling and EMT. Based on this observation, we propose that targeting intracellular acidification could be an effective therapeutic strategy for preventing or slowing the progression of lung cancer metastasis. The findings from our animal study lend further support to this hypothesis. Using a mouse model via tail vein injection, we showed that administering BAF and LAN resulted in varying levels of in vivo metastasis suppression of 889DTC cells (Fig. 7. G–J). This implies that targeting the excessively activated V-ATPase in lung cancer cells, due to reduced FBXO9 expression, could present a potential therapeutic avenue for controlling lung cancer metastasis. Further research should explore interventions targeting the FBXO9-V-ATPase axis for therapeutic purposes. Identifying the specific molecular mechanisms and signaling pathways influenced by FBXO9 could lead to the development of targeted therapies to mitigate lung cancer progression and metastasis.

## Conclusions

In summary, our preclinical study provides substantial evidence for the anti-cancer properties of FBXO9. We demonstrated that FBXO9 plays a crucial role in the regulation of the V-ATPase proton pump by promoting the ubiquitination of the ATP6V1A subunit. FBXO9 inhibits the assembly and function of the V-ATPase complex in the vesicular membrane through this non-degradative ubiquitination process. Consequently, the Wnt signaling pathway and EMT are suppressed in lung cancer cells. These findings shed light on potential novel therapeutic strategies using FBXO9 and highlight its importance in lung cancer treatment.

### Electronic supplementary material

Below is the link to the electronic supplementary material.

Supplementary Material 1

## Data Availability

The data supporting the conclusions of this research are available from the corresponding author upon reasonable request.

## Abbreviations

CRL: Cullin-RING ubiquitin ligase
EMT: Epithelial-mesenchymal transition
FL: Full-length
GEO: Gene Expression Omnibus
H&E: Hematoxylin and eosin
LUAD: Lung adenocarcinoma
LUSC: Lung squamous cell carcinoma
NSCLC: Non-small cell lung cancer
OS: Overall survival
TCGA: The Cancer Genome Atlas
CHX: Cycloheximide
CRISPR: Clustered regularly interspaced short palindromic repeats
FBXO: F-box only protein
IHC: Immunohistochemistry
IP: Immunoprecipitation
LacZ: β-galactosidase gene z
TBST: Tris Buffered Saline with Tween-20
TMA: Tumor tissue microarray
Wnt: Wingless-Type MMTV integration site family
