# Supplementary material for: Ubiquitin ligase subunit FBXO9 inhibits V-ATPase assembly and impedes lung cancer metastasis
Source: Exp Hematol Oncol. 2024 Mar 14;13:32. doi: 10.1186/s40164-024-00497-4 (PMC10938814; doi:10.1186/s40164-024-00497-4)
Supplement: Supplementary file 1 — Supplementary Material 1 [file 40164_2024_497_MOESM1_ESM.docx]

**Supplemental data**

**
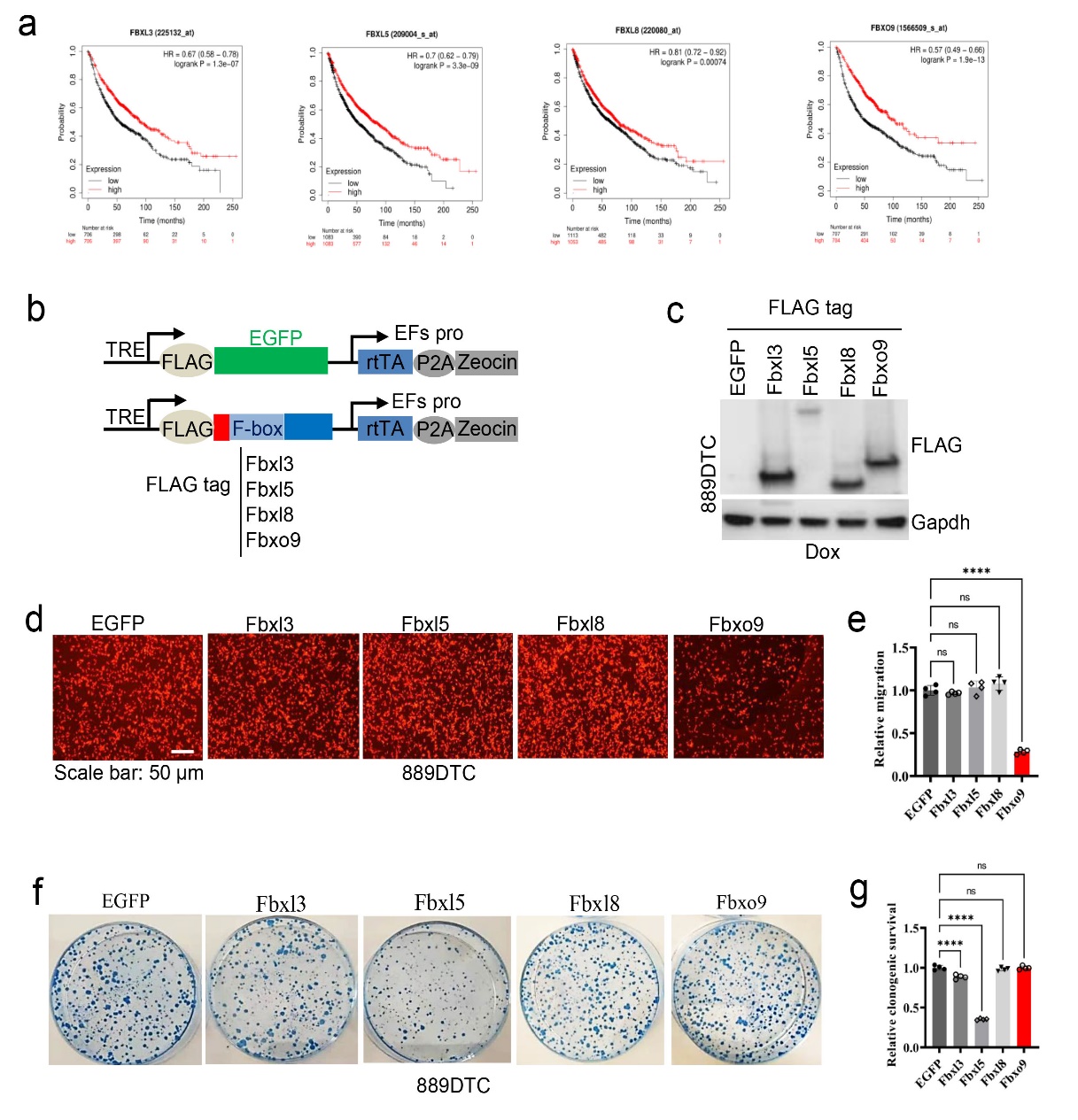
**

**Supplementary Figure 1:** **Effect of Fbxl3, Fbxl5, Fbxl8, and Fbxo9 on cell migration in 889DTC (**related to Figure 1a-e).

**a** KM plotter analysis (kmplot.com/analysis) shows that upregulation of FBXL3, FBXL5, FBXL8, or FBXO9 is associated with improved overall survival in lung cancer patients. **b** Schematic diagram depicting the control and regulated expression of Fbxl3, Fbxl5, Fbxl8, Fbxo9, or EGFP (negative control) in 889DTC cells using the Tet-On system. **c** Immunoblot analysis confirms the expression levels of Fbxl3, Fbxl5, Fbxl8, and Fbxo9 in 889DTC cells treated with Doxycycline. **d, e** Ectopic expression of Fbxl3, Fbxl5, Fbxl8, and Fbxo9 influences 889DTC cell migration, as shown by migration assay (d) and quantification analysis (e). (f, g) Investigation of the influence of ectopic expression of Fbxl3, Fbxl5, Fbxl8, and Fbxo9 on the clonogenic survival of 889DTC cells (f), followed by quantification analysis (g). Statistical significance was denoted as * *P* < 0.05, ** *P* < 0.01, and *** *P* < 0.001.


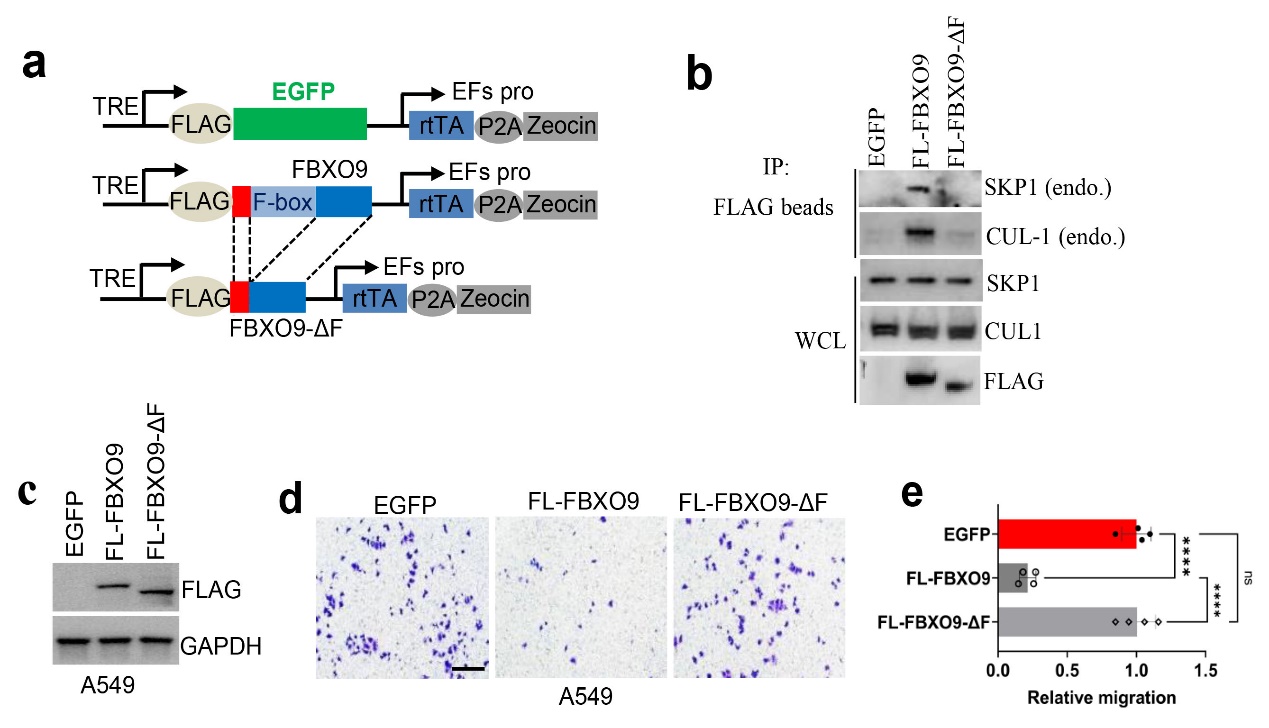


**Supplementary Figure 2: Effect of FBXO9 ectopic expression on A549 cell migration** (related to Supplementary Figure 1d-g).

**a** Inducible expression of FBXO9 and its F-box domain mutant variant in A549 cells using the Tet-on system, as depicted in the schematic diagram. **b** Co-immunoprecipitation assay to determine the binding capacity between FBXO9-ΔF and the SKP1 protein. **c** Ectopic expression of FBXO9 in A549 cells was confirmed by immunoblot analysis after treatment with Doxycycline (1 µg/ml). **d, e** Influence of FBXO9 and FBXO9-ΔF ectopic expression on A549 cell migration, as shown by migration assay (d) and quantification analysis (e). Statistical significance was denoted as * *P* < 0.05, ** *P* < 0.01, and *** *P* < 0.001.


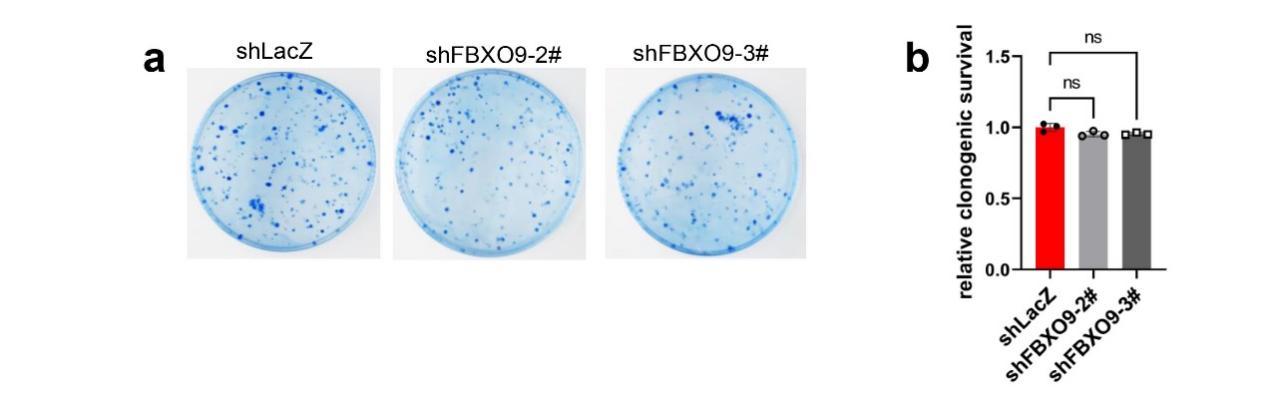


**Supplementary Figure 3: Effect of FBXO9 knockdown on clonogenic survival in H1299 lung cancer cells** (related to Figure 1).

H1299 cells were treated with FBXO9-targeting shRNA to downregulate its expression. The clonogenic survival assay was then performed to evaluate the impact of FBXO9 knockdown on cell viability. Statistical significance was denoted as * *P* < 0.05, ** *P* < 0.01, and *** *P* < 0.001.


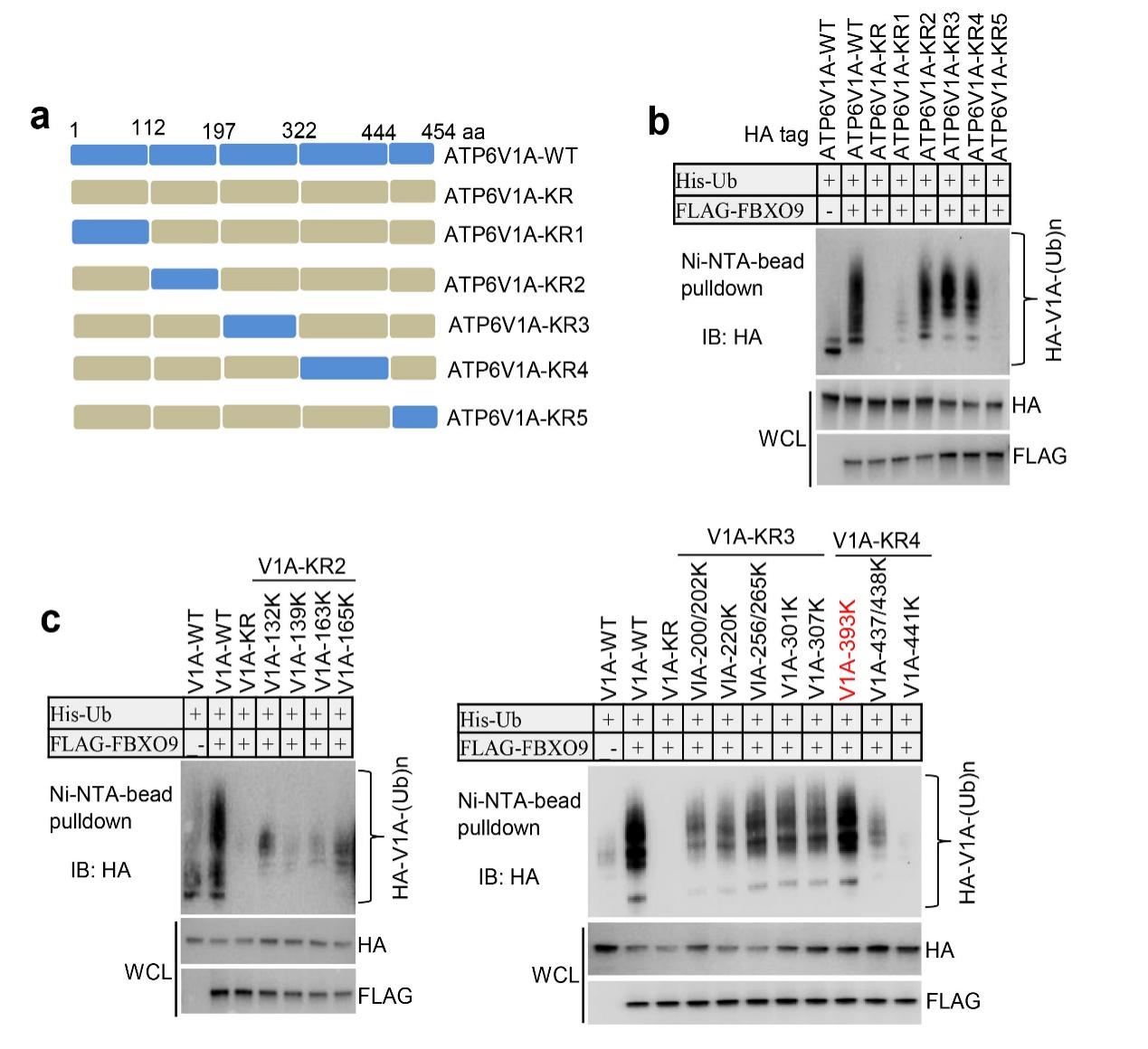


**Supplementary Figure 4: Identification of FBXO9-mediated ubiquitination site(s) on ATP6V1A protein** (related to Figure 4).

**a** Cloning strategy for generating ATP6V1A mutants used to screen for FBXO9-mediated ubiquitination sites. The wild type ATP6V1A (ATP6V1A-WT) was synthesized with all lysine (K) residues replaced by arginine (R), resulting in the ATP6V1A-KR mutant. PCR and seamless cloning techniques were employed to create specific overlapping mutants, namely ATP6V1A-KR2, ATP6V1A-KR3, ATP6V1A-KR4, and ATP6V1A-KR5. **b** Ubiquitination assay performed to determine the domain containing the ubiquitination site in the indicated overlapping mutants upon overexpression of FBXO9. **c** Ubiquitination assay results demonstrating site-specific ubiquitination in ATP6V1A-KR2, ATP6V1A-KR3, and ATP6V1A-KR4 mutants upon FBXO9 overexpression.


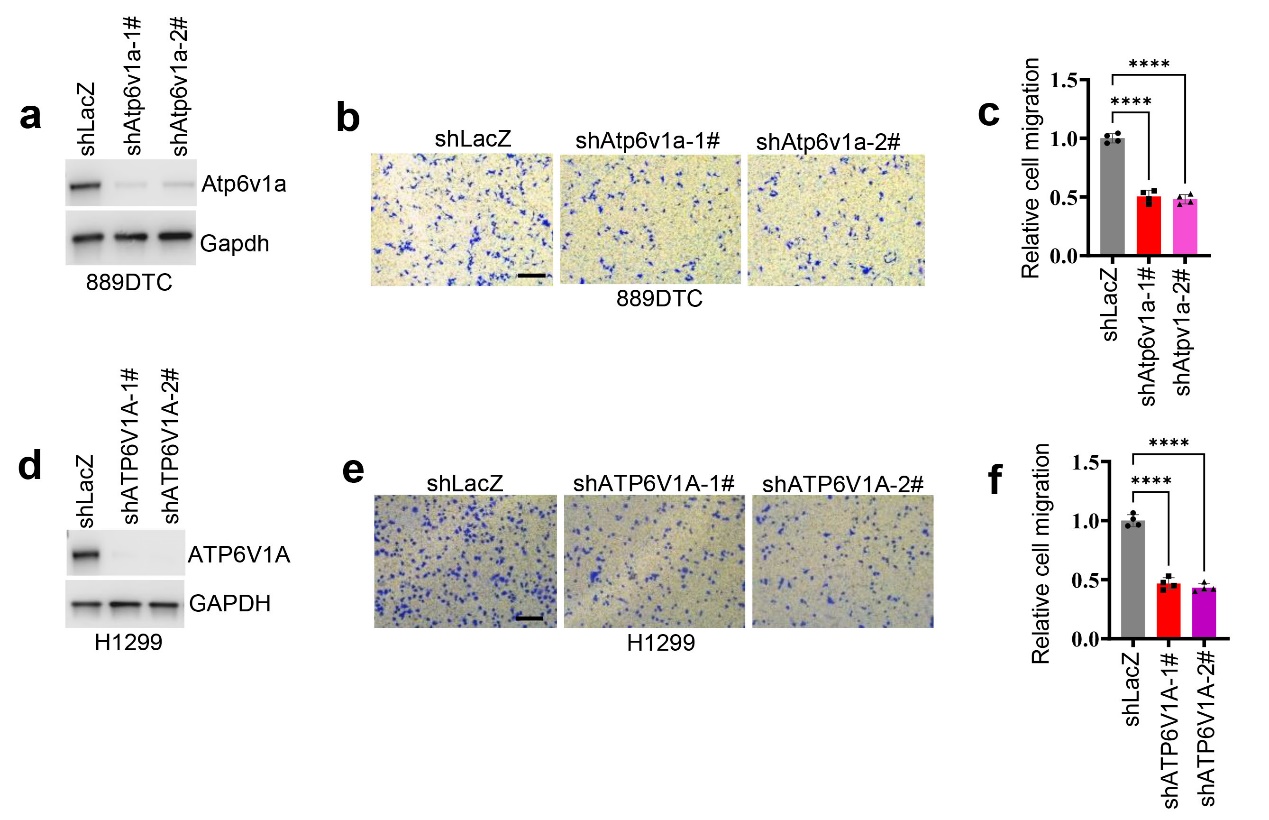


**Supplementary Figure 5: Effect of ATP6V1A downregulation on lung cancer cell migration.**

**a-c** ATP6V1A knockdown in 889DTC cells using shRNA (a), followed by a cell migration assay (b) and quantification (c). **d-f** ATP6V1A knockdown in H1299 cells using shRNA (d), followed by a cell migration assay (e) and quantification (f). Statistical significance was determined as * *P* < 0.05, ** *P* < 0.01, and *** *P* < 0.001.


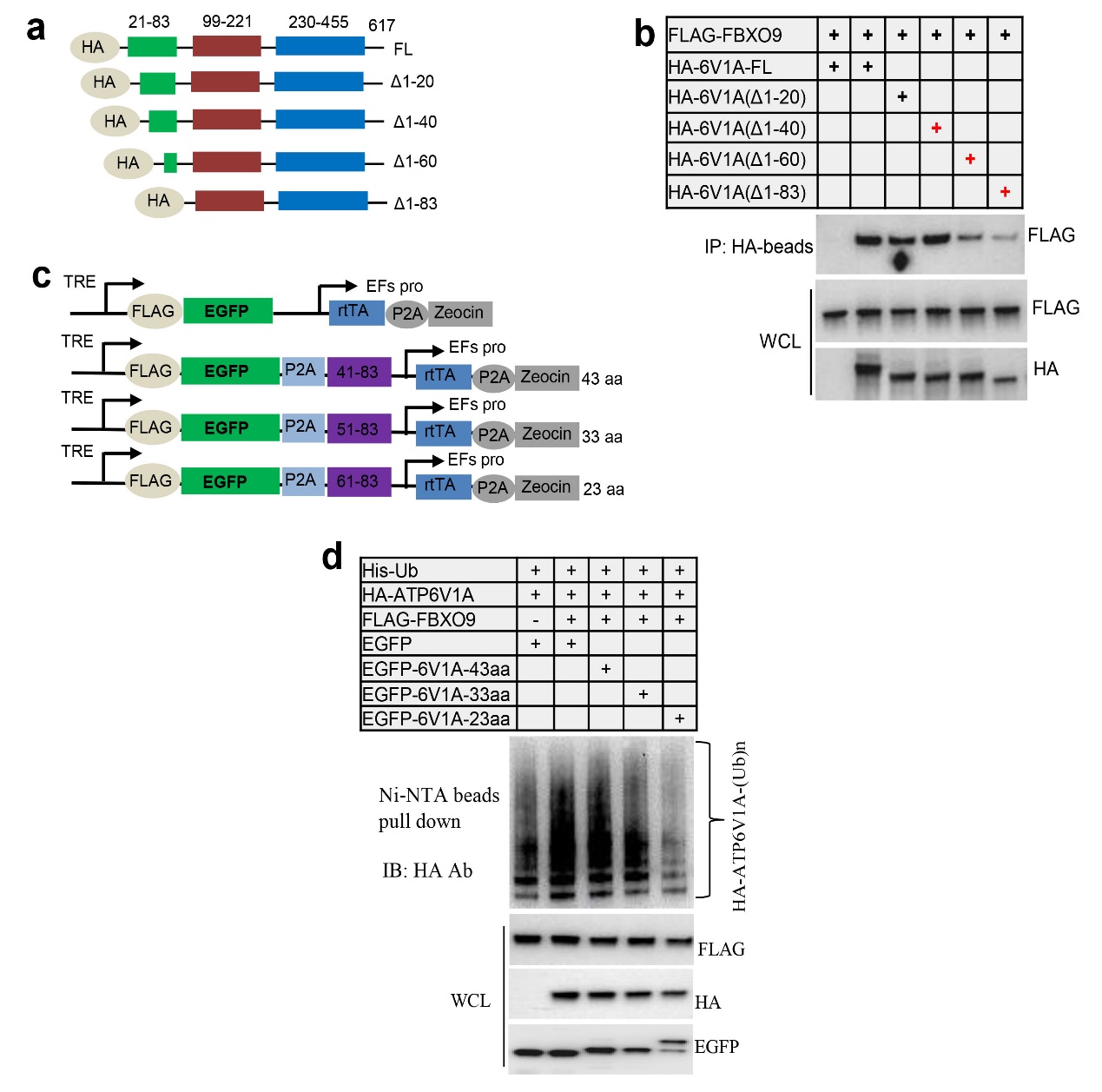


**Supplementary Figure 6: Development of a small peptide inhibitor of ATP6V1A ubiquitination by FBXO9.**

**a** Truncation mutants of ATP6V1A were generated by removing different lengths of the N-terminus region, guided by the importance of amino acid regions 1 to 83 (1-83aa) for interaction with FBXO9 (as shown in Figure 2e). **b** Co-immunoprecipitation experiment performed to identify the specific amino acid region in ATP6V1A that exhibits significant interaction with FBXO9. **c** A schematic diagram depicting the expression of small peptides targeting ATP6V1A, with different lengths and targeting amino acid regions of 40-83. These small peptides are linked to EGFP via the self-cleaving peptide P2A and their expression is regulated by the Tet-on gene expression system. **d** Ubiquitination assay conducted to evaluate the efficacy of the small peptide inhibitor in blocking ATP6V1A ubiquitination by FBXO9. This assay provides insights into the effectiveness of the identified small peptide inhibitor.

**
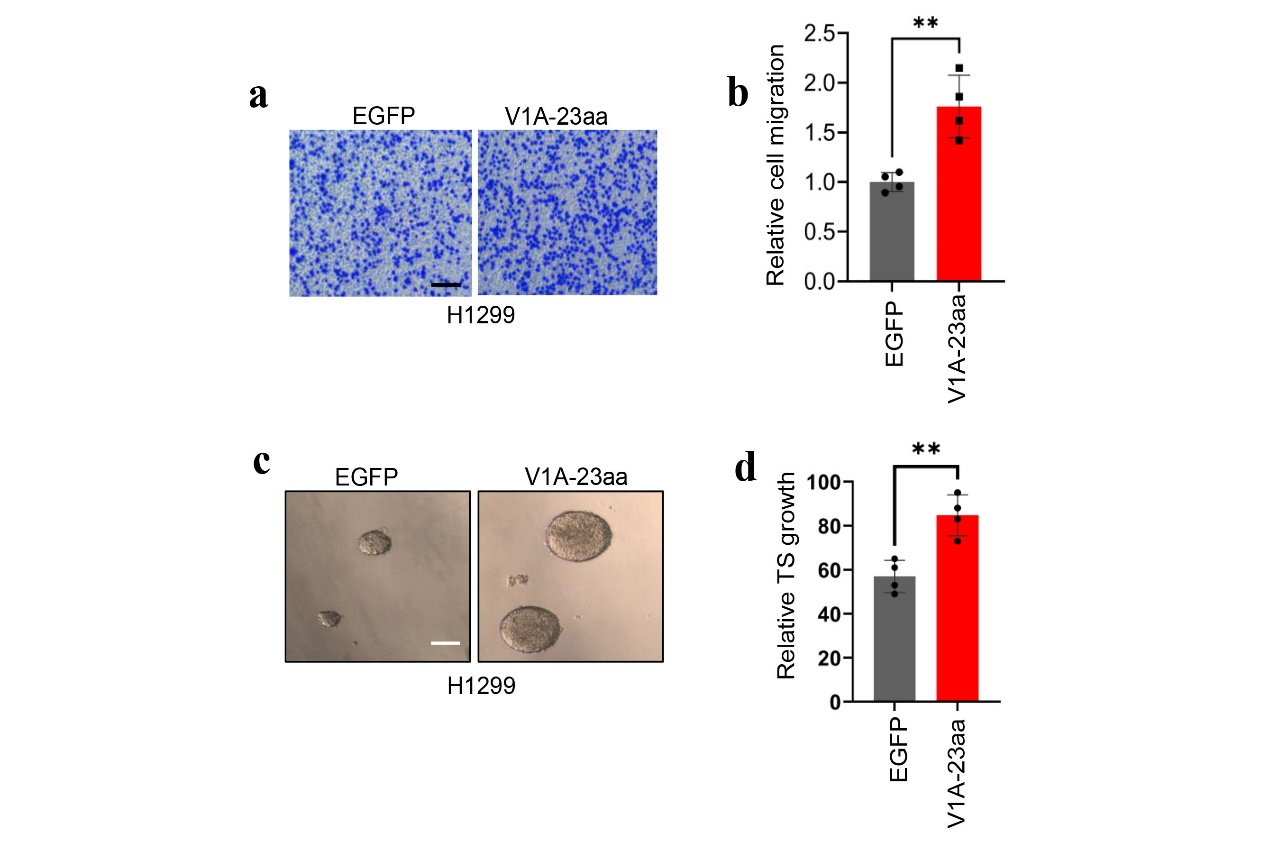
**

**Supplementary Figure 7: Impact of blocking ATP6V1A ubiquitination on H1299 cell migration and tumor sphere formation**.

**a, b** Transwell migration assay and tumor sphere formation assay were performed using H1299 cells expressing V1A-23aa to evaluate the effect of blocking ATP6V1A ubiquitination on cell migration (a) and tumor sphere growth (b). **c, d** Results of the transwell migration assay (c) and tumor sphere formation assay (d) indicated the impact of blocking ATP6V1A ubiquitination on H1299 cell migration and tumor sphere growth. Statistical significance was denoted as * *P* < 0.05, ** *P* < 0.01, and *** *P* < 0.001.


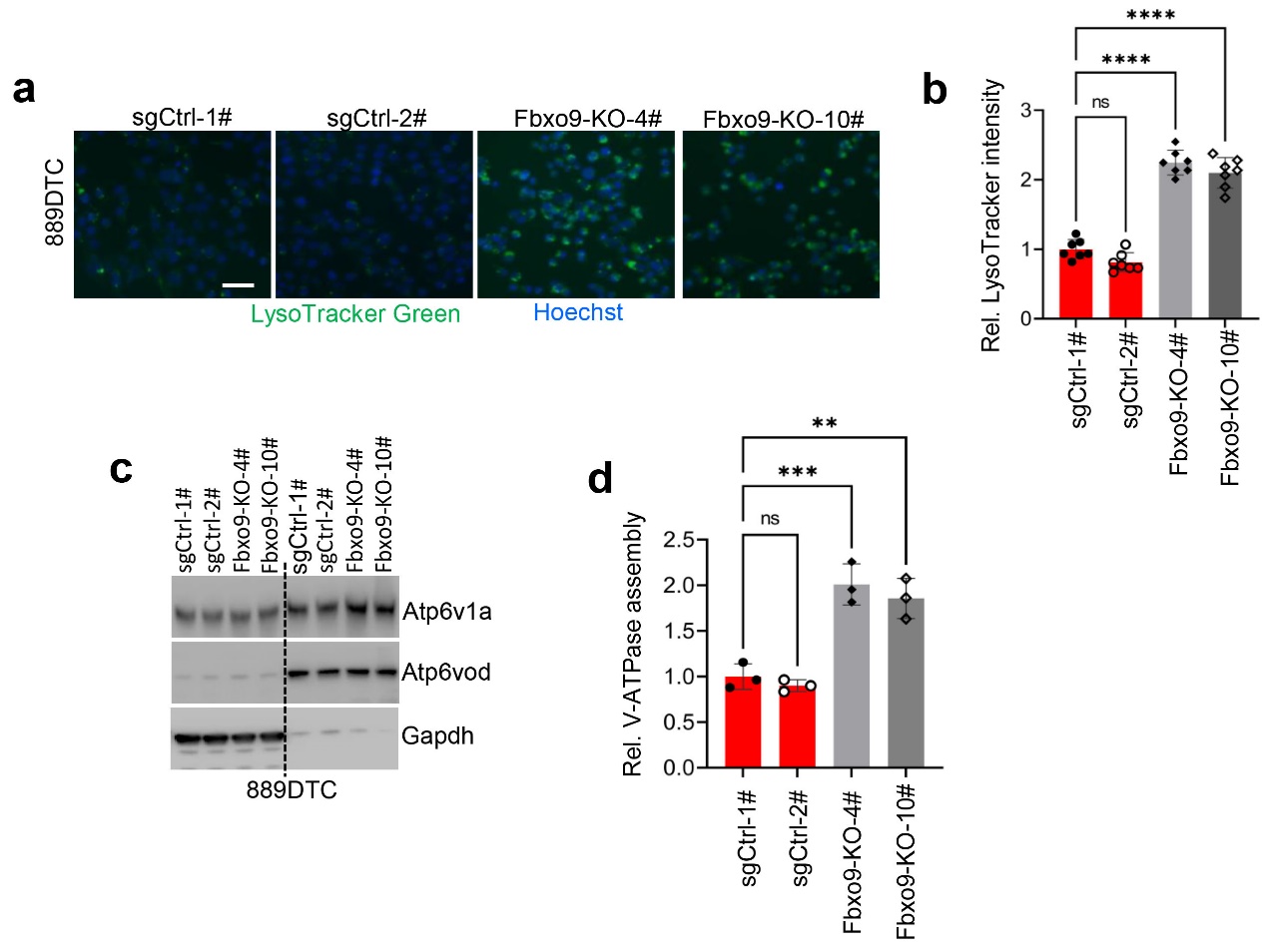


**Supplementary Figure 8: Effect of Fbxo9 knockout on V-ATPase assembly and function in 889DTC cells**

**a, b** Assessment of lysosomal acidity in Fbxo9-knockout 889DTC cells. Representative images show LysoTracker staining in negative control and Fbxo9-knockout cells. Plots depict overall LysoTracker intensity per cell (Scale bar = 100 µm, n = 6-8 images from three independent experiments, *****P* < 0.0001). **c, d** Analysis of V-ATPase assembly in Fbxo9-knockout 889DTC cells. Subcellular fractionation and immunoblot analysis were performed to measure the levels of ATP6V1A and ATP6VoD. The ratio of ATP6V1A to ATP6VoD in the membrane fraction represents the assembly of the V-ATPase (n = 3 independent experiments, ***P* < 0.01,****P* < 0.001).


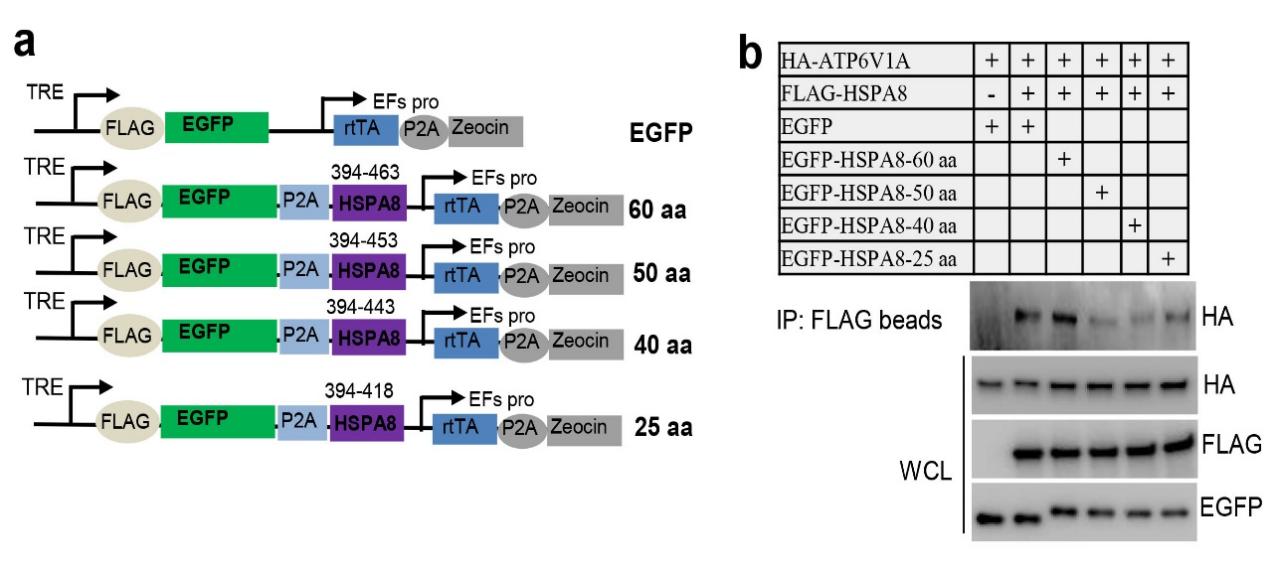


**Supplementary Figure 9. Design and identification of a small peptide blocking interaction between HSPA8 and ATP6V1A.**

To disrupt the interaction between HSPA8 and ATP6V1A, peptides of varying lengths targeting the substrate-binding domain (SBD) of HSPA8 were designed (**a**). Constructs producing 25, 40, 50, and 60 amino acid (aa) length peptides (HSPA8-25aa, HSPA8-40aa, HSPA8-50aa, and HSPA8-60aa) were generated. Immunoprecipitation assays revealed that most of these HSPA8 peptides effectively disrupted the ATP6V1A and HSPA8 interaction in cellular environments, except for HSPA8-60aa peptide (**b**). This experiment demonstrates the design and successful identification of a small peptide capable of blocking the interaction between HSPA8 and ATP6V1A.


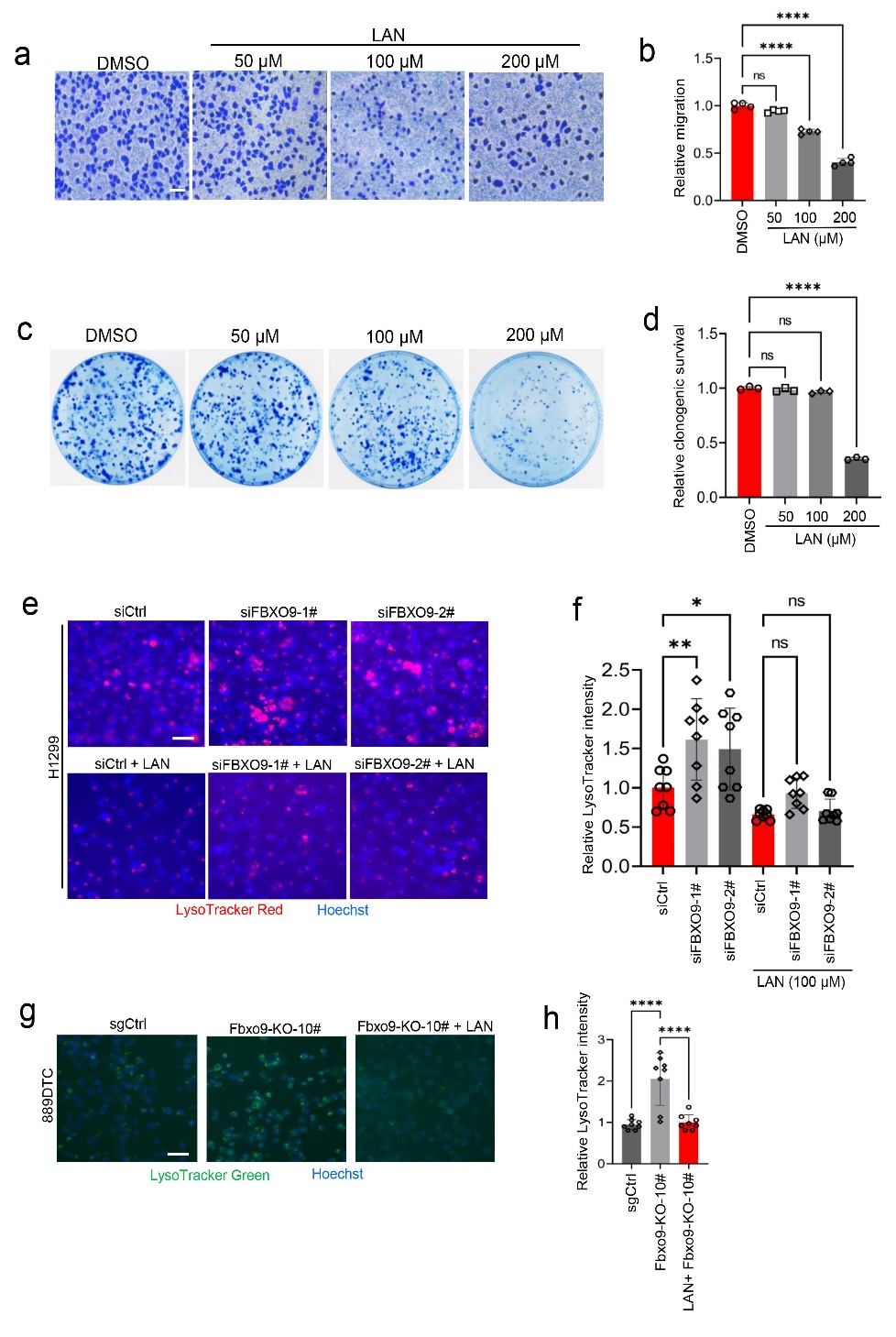


**Supplementary Figure 10: Inhibitory effect of Lansoprazole on V-ATPase in lung cancer cells.**

**a-d** H1299 lung cancer cells were treated with various concentrations of Lansoprazole (LAN) for 12 hours. The impact of the treatment on cell migration and survival was evaluated using transwell migration (a, b) and clonogenic survival (c, d) assays, respectively. Scale bar = 200 µm. **e, f** H1299 cells were transfected with siRNA to deplete FBXO9, followed by treatment with 100 µM Lansoprazole for 12 hours. Lysosomal acidity was assessed by staining with LysoTracker (Red), and the overall intensity of LysoTracker per cell was quantified. Scale bar =100 µm. Data from 6-8 images obtained from three independent experiments are presented. **P* < 0.05, ***P* < 0.01. **g, h** 889DTC cells with Fbxo9 knockout were exposed to 100 µM Lansoprazole for 12 hours. LysoTracker (Green) staining was used to assess lysosomal acidity, and the overall intensity of LysoTracker was measured. Scale bar = 100 µm. Statistical significance was indicated as * *P* < 0.05, ** *P* < 0.01, and *** *P* < 0.001.
